# Aurora A binds to the transactivation domain of c-Myc and recognizes the phosphorylated N-terminal degron motif

**DOI:** 10.1042/BCJ20240726

**Published:** 2025-04-16

**Authors:** Nidhi Joshi, Katie M. Dunleavy, Kaitlin M. Beel, Tiffany A. Engel, Andrew R. Thompson, Felix L. John, David D. Thomas, Nicholas M. Levinson

**Affiliations:** 1 Department of Pharmacology and Masonic Cancer Center, University of Minnesota, 312 Church St. SE, Minneapolis MN 55455, U.S.A.; 2 Department of Biochemistry, Molecular Biology, and Biophysics, University of Minnesota, 312 Church St. SE, Minneapolis MN 55455, U.S.A.

**Keywords:** Aurora A, c-Myc, fluorescence, NMR, protein kinase

## Abstract

The oncoprotein c-Myc is overexpressed or mutated in a large fraction of human cancers. The stability of c-Myc is controlled by phosphorylation of T58 and S62 within a conserved degron motif in the N-terminal transactivation domain (TAD), which triggers recruitment of the SCF ubiquitin ligase. The kinase Aurora A (AurA) has been shown to bind to both c-Myc and its paralog N-Myc and to promote their stability by interfering with ubiquitination and degradation. Here we show, using NMR and Förster resonance energy transfer experiments, that AurA binds to c-Myc through several discrete interactions spanning 145 residues within its TAD. AurA binding to c-Myc is enhanced by phosphorylation of the T58/S62 degron, demonstrating that the kinase recognizes the pool of c-Myc that has been marked for degradation by the ubiquitin proteasome pathway. Although AurA binds to segments of c-Myc flanking the degron, it does not appear to form extensive interactions with the phosphorylated degron itself, potentially leaving it accessible on the AurA surface. These observations establish a foundation for understanding the role of AurA in regulating c-Myc ubiquitination and degradation.

## Introduction

c-Myc is one of the three Myc-family transcription factors (c-Myc, N-Myc, and L-Myc) that play central roles in the pathways coupling extracellular signals to cell proliferation [1,2]. Amplification and dysregulation of Myc transcription factors is a major driver of many cancer types, with c-Myc implicated in perhaps half of all cancers [3]. N- and L-Myc have more restricted expression than c-Myc and are linked to the formation of neuroblastoma and small cell lung carcinomas, respectively [4–6].

Myc family proteins range in length from 364 to 464 residues and contain at their C-terminus a conserved basic helix-loop-helix leucine zipper motif. The leucine zipper segment forms a heterodimer with Myc-associated factor X (Max) to create the functional DNA-binding domain [7,8]. Myc:Max heterodimers recognize enhancer box (E-box) sequences in gene promoters and enhancers [9]. The N-terminal region of Myc is intrinsically disordered and contains six short segments of high sequence conservation, termed Myc boxes (MBs), that are responsible for recruiting transcriptional machinery [10,11] and for binding DNA and chromatin [12,13]. As originally defined [14], the transactivation domain (TAD) of c-Myc spans residues 1–143 and includes MB0, MBI, and MBII, but recent studies suggest that the TAD may extend beyond this [10].

Among the MBs, MBI plays a critical role in controlling Myc protein stability and turnover [15]. MBI contains the key degron motif (residues 56–63) that recruits Myc to the SCF^Fbxw7^ ubiquitin ligase for ubiquitination and subsequent proteasomal degradation. To initiate ubiquitination, the degron is first phosphorylated on S62 by ERK or by cyclin-dependent kinases (CDK1 or CDK2), and subsequently on a second degron residue, T58, by GSK3b [16]. The doubly phosphorylated degron of Myc is recognized by the Fbxw7 subunit of the SCF^Fbxw7^ ubiquitin ligase [17,18], resulting in ubiquitination of Myc and proteasomal degradation. Recently, a second degron motif has been identified in c-Myc (residues 242–249) that is also recognized by the SCF^Fbxw7^ ubiquitin ligase [19] and may co-operate with the primary degron in MBI to promote ubiquitination. However, the kinases responsible for phosphorylation of T244 and T248 in the second degron have yet to be identified, and it is thought that the T58 degron is sufficient for efficient Myc degradation under most conditions [19].

Several studies have shown that the ubiquitination of both N-Myc and c-Myc can be regulated by the protein kinase Aurora A (AurA). AurA binds to N-Myc and c-Myc and is thought to slow the formation of ubiquitin chains by the SCF^Fbxw7^ complex, resulting in enhanced stability of N-Myc in neuroblastoma and neuroendocrine prostate cancer and of c-Myc in liver cancer [20–22]. Immunoprecipitation studies in these cancer cells indicate that AurA is assembled into larger complexes containing both N/c-Myc and Fbxw7 [20], suggesting that the binding of AurA to Myc results in both proteins being incorporated into complexes with ubiquitin ligase components. However, the mechanism by which AurA modulates ubiquitination remains unclear. Interestingly, this role of AurA does not require catalytic activity, as it is unaffected by conventional ATP-competitive AurA inhibitors. In contrast, inhibitors that stabilize the flexible activation loop of the kinase in an inactive conformation can displace AurA from both N-Myc and c-Myc and promote Myc degradation [23,24]. A proteolysis-targeting chimera degrader of AurA also triggers efficient N-Myc degradation in N-Myc-overexpressing neuroblastoma cell lines [25].

A recent NMR study localized the interaction of AurA with N-Myc to the MB0, MBI, and MBII segments [26]. X-ray structures of AurA bound to a short segment of N-Myc show that residues 69–81 downstream of MBI traverse the activation loop of AurA and form an alpha helix that occludes the substrate binding site [27,28]. However, the segment of N-Myc visualized in this structure is not conserved in either c-Myc or L-Myc, and the structural nature of the AurA/c-Myc interaction remains undefined.

Here, we use Förster resonance energy transfer (FRET)-binding measurements and NMR to study how the unstructured region of c-Myc interacts with AurA. We show that the interacting region comprises a 145-residue segment of c-Myc, including MB0, MBI, and MBII. Additionally, we find that dual phosphorylation of the key degron motif on T58 and S62 of c-Myc enhances AurA binding affinity and induces a polyproline type II (PPII) helix conformation in the degron. These results show that AurA recognizes the key phosphorylation events that trigger c-Myc ubiquitination and degradation, underscoring an important role for AurA in the Myc lifecycle.

## Results

### AurA binds to c-Myc via extensive interactions spanning the TAD

**Figure 1:**
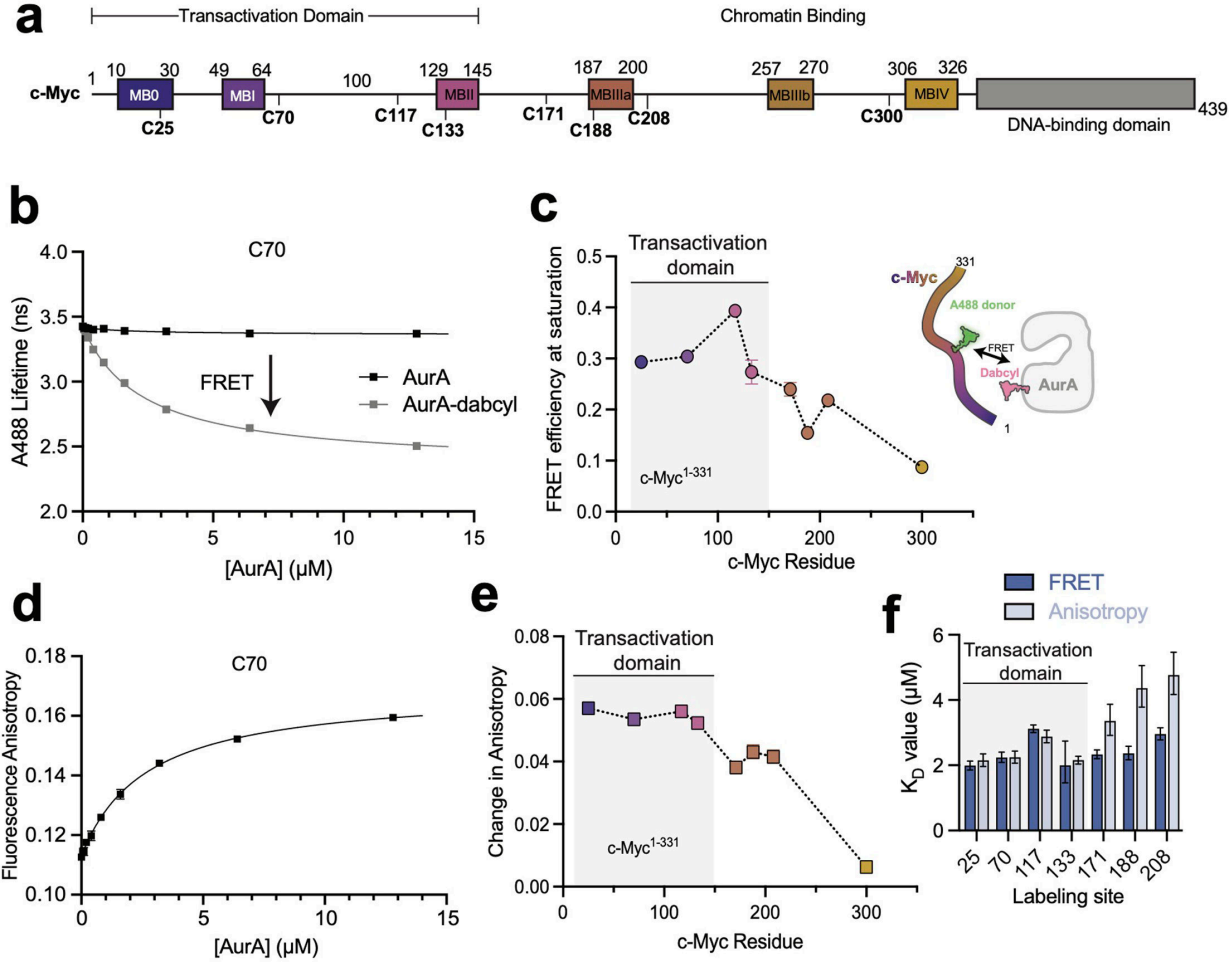
AurA binds to multiple sites across 145 residues of the intrinsically disordered region of c-Myc. **(a**) Schematic representation of the primary structure of c-Myc showing the six Myc boxes within the N-terminal intrinsically disordered region and the C-terminal DNA-binding domain. The locations of the eight native cysteine residues used for incorporating fluorophores are indicated. (**b**) Representative fluorescence lifetime data for c-Myc^1-331^ labeled with A488 on C70, showing titrations of AurA in either unlabeled form (donor-only control experiment) or labeled with dabcyl acceptor dye. Data represent mean values ± s.d.: *n* = 3; one representative experiment of three is shown. (**c**) FRET efficiency values observed at saturating AurA concentrations for different c-Myc^1-331^ constructs labeled at each of the eight cysteine sites. Data represent mean ± 95% CI from binding curve fits; *n* = 3. One representative example of three independent experiments is shown. (**d**) Representative fluorescence anisotropy data for c-Myc^1-331^ labeled with A488 on C70. Data represent mean values ± s.d.: *n* = 3; one representative experiment of three is shown. (**e**) Total change in fluorescence anisotropy observed upon saturation with AurA as measured at each of the eight labeled c-Myc mutants. Data represent mean ± 95% CI from binding curve fits; *n* = 3. One representative experiment of three is shown. (**f**) Comparison of equilibrium dissociation constants for AurA binding to c-Myc determined at different labeling sites by FRET and fluorescence anisotropy (the C300 site was excluded). Values represent the mean ± 95% CI from binding curve fits; *n* = 3. One representative experiment of three is shown. AurA, Aurora A; FRET, Förster resonance energy transfer.

We used FRET experiments to map the interaction between the kinase domain of AurA (AurA^122-403^) and a construct of c-Myc spanning the intrinsically disordered region (c-Myc^1-331^). c-Myc was labeled with the donor dye Alexa 488 (c-Myc-A488) and AurA with dabcyl acceptor (AurA-dabcyl). c-Myc^1-331^ contains eight native cysteine residues distributed across the length of the construct and spanning MB0–MBIV (Figure 1a). For each of these native cysteines, we generated a single-cysteine c-Myc^1-331^ construct by mutating all other cysteines to serine, allowing us to use thiol labeling to selectively incorporate a single A488 dye at any of the eight native sites (referred to hereafter as C25, C70, C117, C133, C171, C188, C208, and C300).

We used a custom-built fluorescence lifetime plate reader [29] to track FRET between c-Myc-A488 and AurA-dabcyl. Titrations of AurA-dabcyl, but not unlabeled AurA, led to large decreases in the fluorescence lifetime at seven of the donor labeling sites and a more modest decrease at C300 (Figure 1b, Supplementary Figure S1). FRET efficiencies calculated at saturation ranged from 27% to 39% for the N-terminal sites near MB0 (C25), MBI (C70), and MBII (C117 and C133) within the canonical TAD [14] but gradually decreased toward the C-terminal end of the construct (C171, C188, C208, and C300) (Figure 1c). Given the Förster radius of 49 Å for the A488/dabcyl dye pair, the average FRET efficiencies for the four N-terminal sites correspond to average FRET distances of 53–58 Å, indicating that multiple regions of the MB0–MBII segment come into close proximity with AurA.

In the same experiments, we also probed AurA binding to c-Myc^1-331^ via the fluorescence anisotropy of the A488 dye, using the donor-only control samples containing unlabeled AurA. Substantial changes in fluorescence anisotropy were observed at the seven N-terminal sites upon AurA binding (Figure 1d, Supplementary Figure S2), whereas the C300 site showed little change, consistent with the lack of a substantial FRET signal at this site. Indeed, the pattern of fluorescence anisotropy changes across the eight cysteines was reminiscent of the observed pattern of FRET efficiencies (Figure 1e), with large changes observed for the sites within the MB0–MBII region, and smaller changes for the more C-terminal sites.

Values for the equilibrium dissociation constants for AurA binding were obtained from the concentration dependence of the FRET and fluorescence anisotropy signals (Figure 1f). For the four N-terminal sites in the MB0–MBII region, the measured *Kd* values were consistent between the two techniques and ranged from 2 to 3 mM depending on the site. These values are similar to those reported previously for AurA binding to N-Myc [27].

Taken together, these data support a model in which the interaction of AurA with c-Myc spans the 145-residue TAD. The high FRET efficiencies observed at C25 (within MB0), C70 (adjacent to MBI), and C117 and C133 (within MBII) are consistent with the formation of protein–protein interactions between c-Myc and AurA within MB0–MBII, and the relatively large changes in fluorescence anisotropy observed at these sites upon AurA binding supports this model. In contrast, the gradual fall-off in FRET efficiency beyond MBII is consistent with the increase in expected separation with polypeptide length predicted by random coil models [30], suggesting that the FRET signals at these C-terminal sites arise not from local interactions but from the same interaction of AurA with the N-terminal TAD.

### Phosphorylation of T58 and S62 within the MBI degron enhances AurA binding

**Figure 2:**
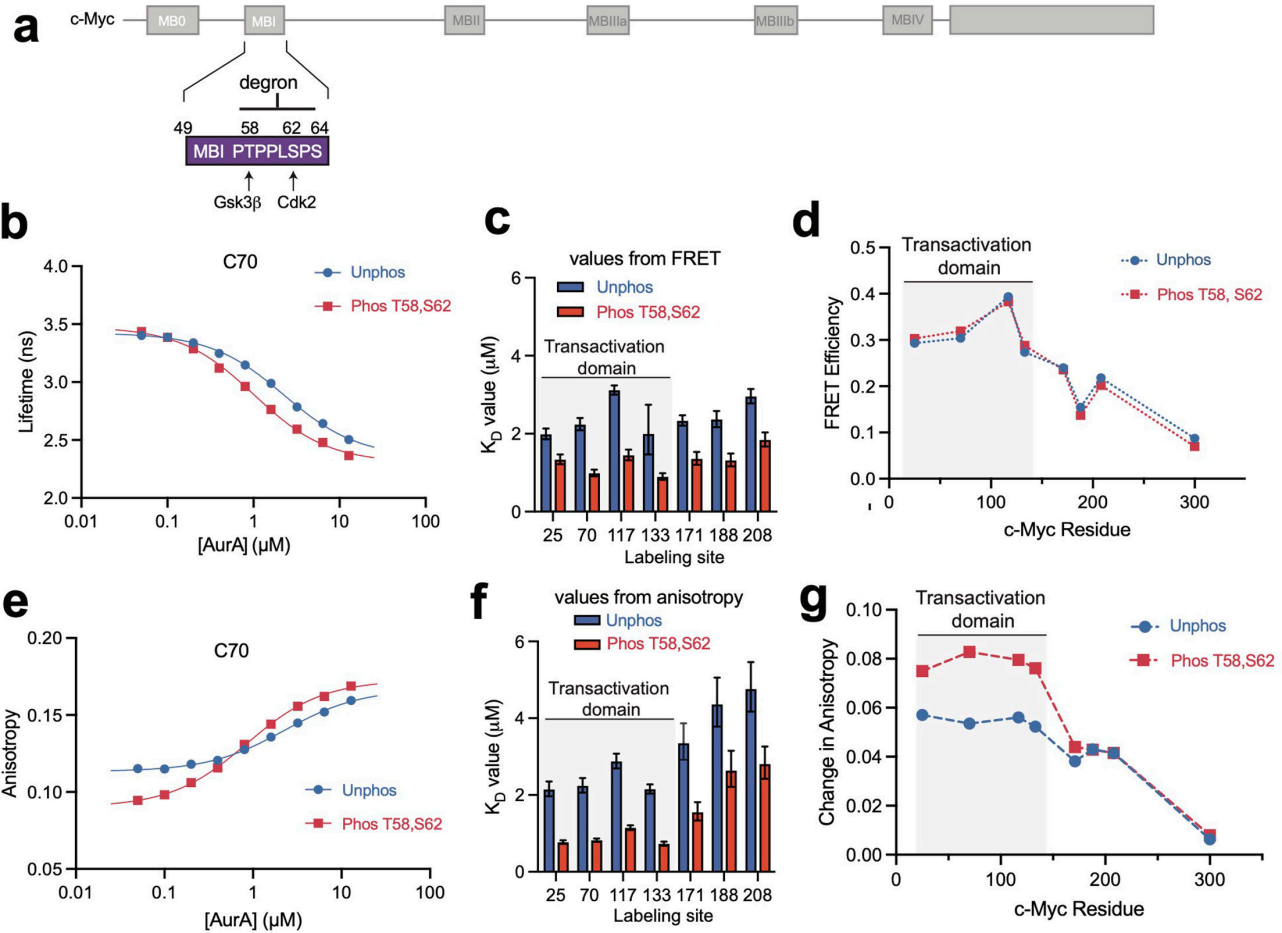
Phosphorylation of T58 and S62 in the c-Myc degron motif enhances AurA binding. **(a**) Schematic representation of MBI within the primary structure of c-Myc, with the degron sequence and the phosphorylation sites shown. (**b**) Representative fluorescence lifetime data for c-Myc^1-331^ labeled with A488 on C70, showing titrations of dabcyl-labeled AurA to unphosphorylated c-Myc (Unphos) or c-Myc phosphorylated on T58 and S62 (Phos T58,S62). Data represent mean values ± s.d.: *n* = 3; one representative experiment of three is shown. (**c**) Equilibrium dissociation constants for AurA binding to phosphorylated and unphosphorylated c-Myc^1-331^ as determined by FRET experiments at the different labeling sites (C300 was excluded), with unphosphorylated c-Myc samples shown in blue and phosphorylated samples shown in red. Values represent mean ± 95% CI from binding curve fits; *n* = 3. One representative experiment of three is shown. (**d**) FRET efficiency values observed at saturating AurA concentrations for each labeled c-Myc mutant in unphosphorylated (Unphos) and phosphorylated (Phos) forms. Values represent mean ± 95% CI from binding curve fits; *n* = 3. One representative experiment of three is shown. (**e**) Representative fluorescence anisotropy binding data using phosphorylated (Phos) and unphosphorylated (Unphos) c-Myc^1-331^ labeled with A488 on C70. Data represent mean values ± s.d.: *n* = 3; one representative experiment of three is shown. (**f**) Equilibrium dissociation constants for AurA binding to c-Myc mutants as measured at the different labeling sites by fluorescence anisotropy (C300 was excluded), with unphosphorylated c-Myc samples shown in blue and phosphorylated samples shown in red. Values represent mean ± 95% CI from binding curve fits; one representative experiment of three is shown. (**g**) Total change in fluorescence anisotropy observed upon AurA binding as measured with each of the eight labeled c-Myc^1-331^ mutants with unphosphorylated (Unphos) and phosphorylated (Phos T58,S62) forms. Data represent mean ± 95% CI from binding curve fits; one representative experiment of three is shown. AurA, Aurora A; FRET, Förster resonance energy transfer; MB, Myc box.

The primary phosphodegron for controlling c-Myc turnover lies within MBI [16] (residues 49–64) close to the C70 labeling site (Figure 2a). To test whether AurA binding is affected by phosphorylation of the degron, we performed *in vitro* phosphorylation reactions, using Cdk2:cyclinA to phosphorylate S62 [31] and Gsk3b to phosphorylate T58. Dual phosphorylation was performed for both the c-Myc^1-331^ constructs described above, as well as a shorter c-Myc^1-88^ construct containing only MB0 and MBI. Mass spectrometry confirmed phosphorylation on both T58 and S62 in both cases (Supplementary Figure S3). For the c-Myc^1-331^ constructs, some secondary phosphorylation sites were observed within the C-terminal portion outside the AurA-interacting region, but for the shorter c-Myc^1-88^ construct that lacks this region intact protein LC-MS and tandem LC-MS/MS confirmed that dual T58,S62 phosphorylation was stoichiometric and homogeneous (Supplementary Figure S3i).

Fluorescence anisotropy experiments performed with the homogeneously phosphorylated c-Myc^1-88^ construct labeled with A488 on C70 showed that while phosphorylation of S62 alone had little effect on AurA binding, dual phosphorylation of both T58 and S62 led to a three-fold enhancement of binding affinity (Supplementary Figure S4). An approximately two-fold enhancement in binding affinity was also observed in FRET experiments with T58,S62-phosphorylated c-Myc^1-331^ (Figure 2b). This enhancement was detected at all four labeling sites within the TAD, as well as at more C-terminal sites (Figure 2c).

Despite enhancing the binding affinity of AurA, dual phosphorylation of the degron produced little change to the overall pattern of FRET efficiencies observed at saturation at each labeling site (Figure 2d, Supplementary Figure S1), indicating that the interaction of AurA with the TAD of c-Myc is minimally affected. Fluorescence anisotropy experiments confirmed the enhancement in binding affinity arising from dual phosphorylation of the degron (Figure 2e and f) as well as a similar pattern of anisotropy changes observed at each site at saturation (Figure 2g, Supplementary Figure S2). Note that the differences in anisotropy change for the first four labeling sites arise from lower baseline anisotropies for free phosphorylated versus free unphosphorylated c-Myc (Figure 2f), rather than from major differences in the bound state. Taken together, these data indicate that dual degron phosphorylation enhances AurA binding affinity without triggering a global rearrangement of the c-Myc/AurA interface.

### NMR experiments reveal that AurA binds to sites within MB0 and MBI flanking the c-Myc degron

**Figure 3:**
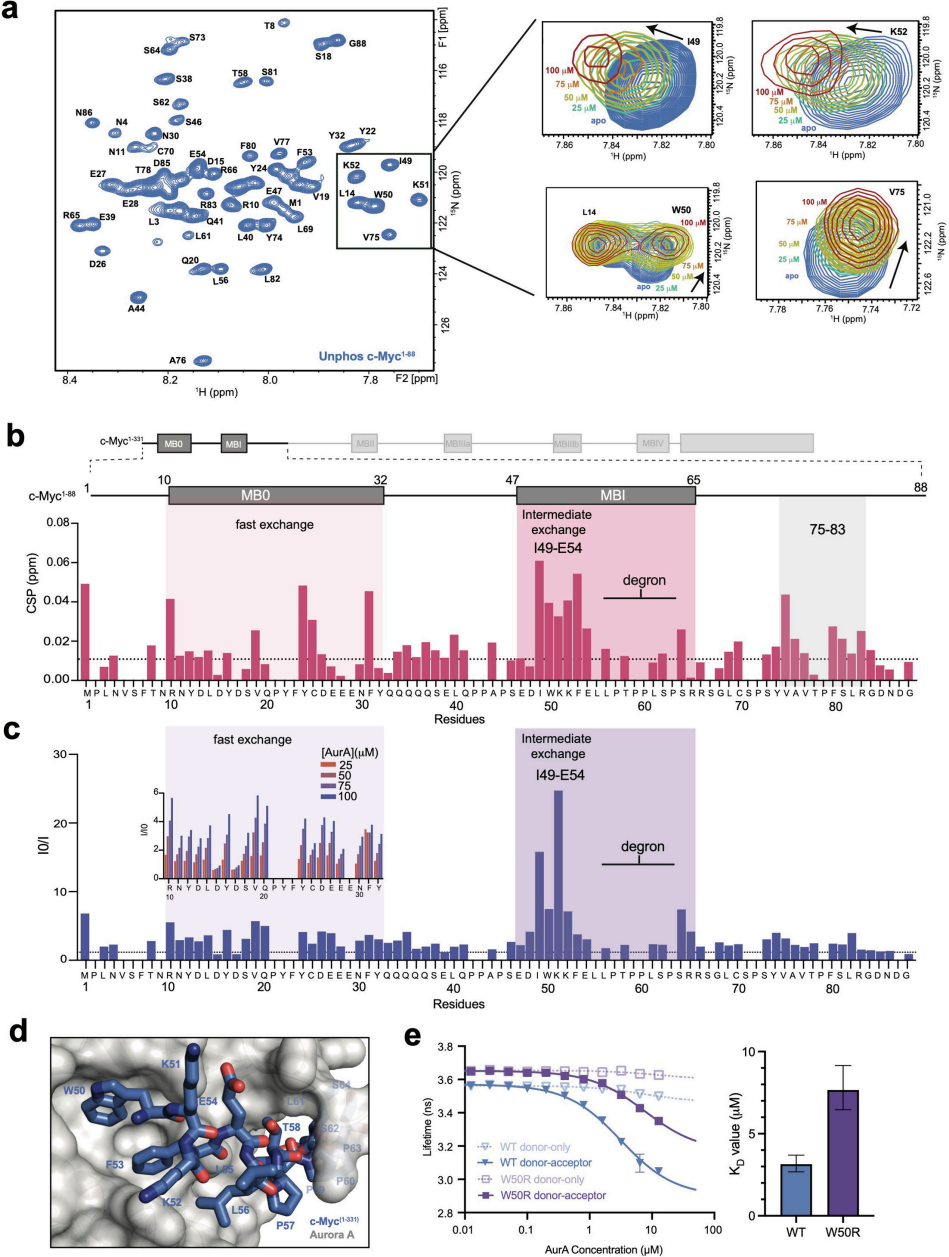
NMR experiments reveal that AurA binds to sites within MB0 and MBI flanking the c-Myc degron. (**a**) Two-dimensional [^1^H, ^15^N]-HSQC spectrum of 100 mM unphosphorylated c-Myc^1-88^ in the absence of AurA (blue). The inset highlights specific resonances that exhibit significant signal loss and/or chemical shift perturbations (CSPs) upon addition of AurA at 25 µM (green), 50 µM (pale yellow), 75 µM (light orange), and 100 µM (dark red). (**b**) A representative schematic showing all the Myc boxes (MBs) across the c-Myc protein, with MB0 and MBI regions highlighted on the CSP profile to emphasize their involvement in binding interactions. The CSP profile of c-Myc^1-88^ upon binding to AurA at a 1:1 ratio is plotted against residue numbers, with the MB0 and MBI segments highlighted by light pink shading. The location of the degron sequence is also indicated. The dashed line represents the average CSP value at the 25 µM concentration of AurA. (**c**) Fold intensity reduction in assigned resonances in the HSQC spectrum of c-Myc^1-88^ upon binding to AurA. Intensity changes, represented as I₀/I ratios, are plotted by residue number, with values greater than 1 representing signal loss. MB0 and MBI are highlighted by light blue shading. All data correspond to a 1:1 ratio of c-Myc^1-88^ and AurA. The dashed line represents the average I₀/I value at the 25 µM concentration of AurA. The inset illustrates a comparative analysis of fold intensity changes for unphosphorylated c-Myc at residues R10-Y32 within the MB0 region, measured across four distinct AurA concentrations. The color coding indicates the respective concentrations of AurA: 25 µM (light orange), 50 µM (light purple), 75 µM (dark purple), and 100 µM (dark blue). These data showcase the concentration-dependent effect of AurA on the intensity changes in the MB0 region of c-Myc. (**d**) The AlphaFold model shows a representative model, with AurA^122-403^ displayed as a gray-colored surface representation and c-Myc (W50-S64) shown in blue stick representation. (**e**) Fluorescence lifetime experiments measuring AurA binding to WT (blue) or W50R (purple) unphosphorylated c-Myc^1-88^ by FRET. Raw lifetime data are shown on the left. Data represent mean values ± s.d.: *n* = 3; one representative experiment of three is shown. *Kd* values are shown on the right. Data represent mean ± 95% CI; one representative experiment of three is shown. AurA, Aurora A; CSP, chemical shift perturbation; FRET, Förster resonance energy transfer; MB, Myc box.

To study the interaction of AurA with c-Myc in greater detail, we used NMR spectroscopy with the c-Myc^1-88^ construct, for which the backbone resonance assignments were previously reported [31,32]. We titrated unlabeled AurA kinase domain into deuterated and ^15^N-labeled c-Myc^1-88^, measured ^1^H-^15^N HSQC spectra (Figure 3a), and tracked the resulting chemical shift perturbations (CSPs) and intensity changes for each resonance as a function of AurA concentration (Figure 3a-c). Addition of AurA led to a deterioration of the HSQC spectra, with a two-fold reduction in average peak intensity observed at a 1:1 stoichiometry (Supplementary Figure S5a,b). This is consistent with a longer rotational correlation time for the AurA:c-Myc complex compared with free c-Myc and with chemical exchange on the μs/ms timescale between bound and free states of c-Myc.

CSPs were observed in two regions of c-Myc^1-88^ spanning residues R10-F31 within MB0 and I49-E54 within MBI but were particularly prominent in the MBI segment (Figure 3b and Supplementary Figure S5a). The observation that AurA binding is primarily localized to the Myc-box segments is consistent with previously reported interactions of AurA with N-Myc [26,27] and reinforces that the interaction with AurA is a conserved feature of these proteins. However, a few CSPs were also detected within a C-terminal segment of c-Myc^1-88^ comprising residues V75-R83 (Figure 3a and b, Supplementary Figure S10) within an insertion that is not present in N-Myc, presumably reflecting interactions unique to c-Myc. Overall, the NMR results are consistent with the high FRET efficiencies observed in the FRET experiments at the C25 labeling site, which lies within MB0, and at the C70 labeling site, which is close to MBI.

The strongest peak intensity changes observed upon addition of AurA were localized to residues I49–E54 within the MBI segment of c-Myc^1-88^ (Figure 3c), consistent with the strong CSPs observed at these positions and suggesting that these residues of c-Myc represent the focal point of the binding interaction. Specifically, residues I49 and K51 underwent a 16- and 25-fold reduction in signal at stoichiometric AurA concentrations, respectively. This indicates that the binding of AurA to this segment occurs in the intermediate exchange regime, resulting in severe line broadening and loss of signal. Reductions in peak intensities were also observed for many residues within MB0, although the degree of attenuation was smaller than observed for MBI. The smaller intensity changes combined with substantial CSP effects are consistent with MB0 binding in the fast exchange regime, unlike MBI. The distinct exchange regimes for the neighboring MBs, coupled with the weaker CSP and intensity effects of MB0 compared with MBI, may indicate that the interaction of MB0 with AurA is more transient than that of MBI. Notably, despite the fact that the strongly affected residues I49–E54 in MBI are immediately upstream of the degron sequence, the residues of the degron itself, including T58 and S62, were not substantially affected by AurA binding in these experiments (Figure 3b and c), indicating that AurA does not interact directly with the degron of unphosphorylated c-Myc.

We used AlphaFold 3 [33] to model the interaction between c-Myc^1-331^ and AurA^122–403^. Three independent modeling runs were performed, resulting in a total of 15 models (Supplementary Figure S13). As expected for an intrinsically disordered protein, the majority of the c-Myc polypeptide chain in these models adopted an array of dissimilar conformations characterized by low confidence, but for a short stretch of seven to nine residues within the MBI segment, the results were more consistent. Specifically, in 12 of the 15 models generated, a segment centered on residues D48–L55, with slight variation between models, adopted an alpha helical conformation and docked into a hydrophobic pocket on the surface of the N-terminal lobe of AurA referred to as the PDK1-interacting fragment (PIF) pocket [34] (Figure 3d, Supplementary Figure S13). This pocket serves as a critical binding site for the AurA activator Tpx2 [35]. Remarkably, the boundaries of the predicted AurA-interacting helix correspond closely to the region where the strongest CSP and intensity effects are observed in our NMR data (residues I49-E54, Figure 3a and b). A prominent feature of all 12 models is that the W50 residue of c-Myc is inserted into the PIF pocket of AurA and buried in the interface. The importance of this residue was confirmed in FRET experiments where the W50R mutation was shown to substantially reduce the binding of c-Myc^1-88^ to AurA (Figure 3e).

### AurA recognizes phosphorylation of T58 within the c-Myc phosphodegron

To investigate how degron phosphorylation affects the interaction of c-Myc with AurA, we performed NMR experiments on c-Myc^1-88^ protein samples that had been phosphorylated on T58 and S62. The backbone resonance assignments of the unphosphorylated c-Myc^1-88^ construct [32] were transferred to the doubly phosphorylated form using standard triple resonance approaches (HNCA, HNCACB, HNCO, CBCA(CO)NH, HN(CA)CO, see Methods) (Supplementary Figure S6a-c). Resonances were assigned for 69 of the 77 non-proline residues in the doubly phosphorylated c-Myc^1-88^ construct, including all non-proline residues in the phosphodegron sequence (residues L56–P63).

Phosphorylation of T58 and S62 produced substantial changes to ^1^H-^15^N HSQC spectra of free c-Myc^1-88^, including an increase in overall signal intensity, the appearance of several new peaks, and notable resonance shifts for several remaining peaks (Supplementary Figure S6a). In particular, the resonances corresponding to T58 and S62 undergo large downfield shifts upon phosphorylation of 0.83 and 0.31 ppm Δδ H^N^, respectively (Supplementary Figure S7). Previous NMR studies have shown that the phosphorylation of proline-rich peptides containing threonine and serine residues triggers strong downfield shifts, with average values of 1.1 and 0.4 ppm reported for phosphothreonine and phosphoserine, respectively [36]. The shifts are thought to arise from stabilization of the PPII helix conformation, which promotes hydrogen bonding between the phosphate group and the backbone NH group of the phosphoserine or phosphothreonine.

**Figure 4:**
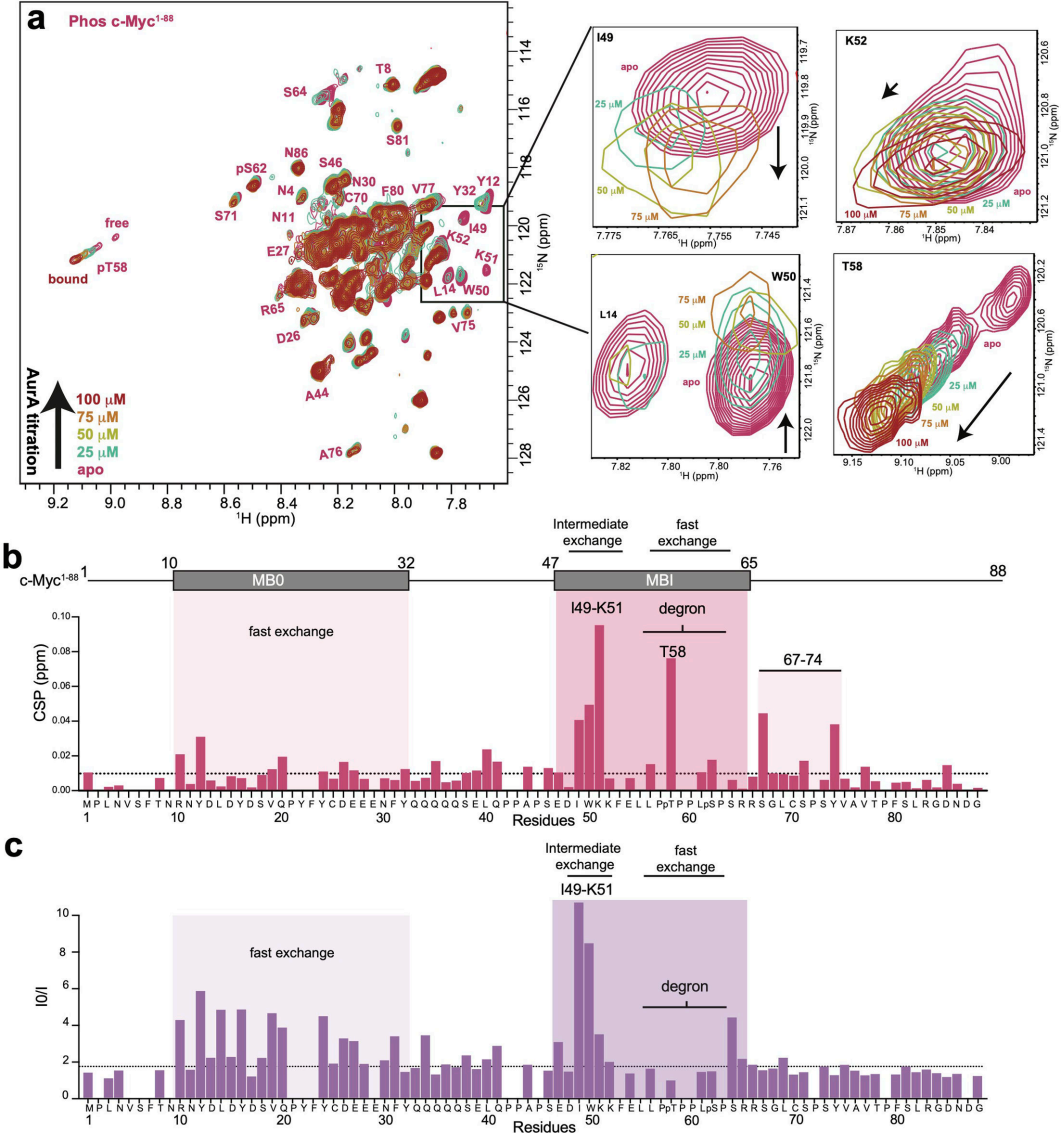
AurA recognizes phosphorylation of T58 within the c-Myc phosphodegron. (**a**) Overlay of [^1^H, ^15^N]-Heteronuclear Single Quantum Coherence (HSQC) spectra of double-phosphorylated c-Myc^1-88^ (shown in magenta) with increasing concentrations of AurA (25 µM to 100 µM). The enlarged views (I49, K52, L14, T58) highlight significant signal loss and/or chemical shift perturbations with increasing AurA concentration. Color coding represents AurA concentrations: 25 µM (green), 50 µM (pale yellow), 75 µM (light orange), and 100 µM (dark red). (**b**) CSP profile of c-Myc^1-88^ at a 1:0.5 ratio with AurA. CSP values are plotted by residue, with the MB0 and MBI segments highlighted by light pink shading, and the degron sequence also marked. The dashed line represents the average CSP value at the 25 µM concentration of AurA. (**c**) Fold intensity changes of c-Myc^1-88^ resonances at a 1:0.5 ratio with AurA, plotted by residue, with values greater than 1 representing signal loss. MB0 and MBI are highlighted by purple shading. The dashed line represents the average I₀/I value at the 25 µM concentration of AurA. AurA, Aurora A; CSP, chemical shift perturbation; MB, Myc box.

To assess how degron phosphorylation affects the interaction with AurA, we titrated unlabeled AurA kinase domain into labeled and doubly phosphorylated c-Myc^1-88^ (Figure 4a). As with unphosphorylated c-Myc (Figure 3b), the CSPs induced by AurA binding were strongest in the MBI region, with residues I49–K51 particularly affected (Figure 4a and b), and although CSPs were also observed in the MB0 segment, they were small in comparison with MBI (Figure 4b and Supplementary Figure S8a). Unexpectedly, the phosphorylated pT58 residue exhibited a dramatic linear chemical shift trajectory upon addition of increasing AurA concentrations (Figure 4a and b and Supplementary Figure S8a). While the pS62 resonance also shifted upon addition of AurA, it was to a much smaller extent than pT58, and the three other non-proline residues within the degron (E54, L56, and L61) did not exhibit measurable CSPs. This suggests that the perturbations observed for the pT58 resonance do not arise from an extensive interaction of AurA with the phosphodegron.

As with unphosphorylated c-Myc^1-88^, the most striking reductions in resonance intensities upon adding AurA to phosphorylated c-Myc^1-88^ were localized to the N-terminal portion of MBI (I49-K51), again consistent with the binding of this important segment occurring in the intermediate exchange regime (Figure 4c and Supplementary Figure S8b). In contrast, despite the large linear chemical shift trajectory observed for the pT58 resonance, neither the pT58 nor the pS62 resonances were attenuated by AurA binding, pointing to a fast exchange regime (Figure 4c and Supplementary Figure S8b). Although intensity changes observed for MB0 were relatively small compared with MBI, they agreed strikingly well with those observed for unphosphorylated c-Myc, indicating that the interaction of AurA with MB0 is minimally affected by degron phosphorylation (Supplementary Figure S11).

Given the limited extent of CSP and intensity effects for the degron segment, the striking linear chemical shift trajectory observed for the phosphorylated pT58 residue is surprising. One possibility is that it arises from a direct interaction with AurA, the extent of which is obscured by the relative paucity of NMR probes in the proline-rich degron segment. We noted, however, that the linear chemical shift trajectory observed for pT58 upon AurA binding was in a similar direction to the downfield shift triggered by phosphorylation of T58, which is driven by induction of PPII helix character, as described above. Thus, an alternative explanation is that the striking effects of AurA on the pT58 resonance do not arise from a direct interaction of the kinase with the phosphodegron but instead occur because the primary interaction of AurA with the central I49-K52 segment of c-Myc further stabilizes the neighboring phosphodegron in the PPII conformation. This interpretation is consistent with the lack of substantial CSPs for the other residues of the degron (Figure 4b) and with the contrasting exchange kinetics for the pT58 and pS62 resonances (fast exchange) compared with the adjacent I49-K52 segment (intermediate exchange). If the latter interpretation is correct, the phosphodegron may be both solvent-exposed and stabilized in the PPII conformation when c-Myc is bound to AurA. This could potentially facilitate the binding of Fbxw7, which tends to bind substrates adopting the PPII conformation [37].

## Discussion

A considerable body of published work has established that AurA has a role in regulating the stability of N-Myc [20,23,24] and has provided biochemical and structural validation of the interaction between the two proteins [26–28]. These studies did not, however, determine whether the degron element, which is fully conserved between N-Myc and c-Myc, participates in binding, and little is known about the interaction between AurA and c-Myc. Here, we show that AurA interacts extensively with elements of c-Myc spanning 145 residues of its unstructured N-terminal region. This region encompasses three different MBs (MB0–MBII) with functions in transcriptional regulation and protein stability [10]. Remarkably, dual phosphorylation of c-Myc on T58 and S62 within the degron segment of MBI enhances the binding affinity of c-Myc for AurA, indicating that AurA recognizes the population of c-Myc molecules that have been marked for degradation via degron phosphorylation. This model is consistent with reports of AurA forming complexes with phosphorylated c-Myc in cells [21] as well as with the SCF ubiquitin ligase subunit Fbxw7 [20].

In addition to MBI, our FRET experiments suggest that AurA interacts directly with both MB0 and MBII of c-Myc, and in the case of MB0, this is corroborated by our NMR data. A recent NMR study on N-Myc is consistent with our findings and further confirmed an interaction of AurA with MBII [26]. While the central role of MBI in controlling c-Myc turnover is established, MB0 and MBII have reported roles in transcriptional regulation rather than in c-Myc stability [10]. These interactions may therefore reflect functions of AurA in transcriptional regulation, similar to those reported for N-Myc, where AurA binding competes with transcriptional complexes including TFIIIC [38]. The absence of detectable binding at the C-terminal regions of c-Myc, including MBIIIb and MBIV, which are associated with chromatin [12] and DNA [13] binding, may reflect a delineation between the transcription-related and chromatin-binding functions of c-Myc.

Our work establishes a biochemical basis for the recognition of c-Myc by AurA. It remains unknown how AurA modulates ubiquitination of c-Myc and N-Myc. In particular, direct recognition of the phosphorylated T58 residue by AurA would appear to conflict with the reported incorporation of AurA into c-Myc/Fbxw7 complexes in cells [20,21], since Fbxw7 binds the phosphorylated degron of c-Myc with 20 nM affinity [19] and would be expected to displace the weaker binding AurA. However, our NMR data suggest an alternative interpretation in which AurA binds upstream of the degron segment and recognizes T58 phosphorylation indirectly through its conformational effects, specifically the promotion of PPII structure in the degron. Such indirect recognition may leave the phosphodegron accessible when c-Myc is bound to AurA, allowing Fbxw7 binding to be accommodated and AurA to be recruited into c-Myc/SCF^Fbxw7^ complexes. The subsequent steps that lead to modulation of c-Myc ubiquitination by AurA remain unknown and will be the subject of future studies.

## Methods

### Protein production

The c-Myc constructs (c-Myc^1-88^ and c-Myc^1-331^) were expressed from a pET28 vector in BL21(DE3) *Escherichia coli* cells and contained a non-cleavable N-terminal His-tag. Mutagenesis of c-Myc constructs to introduce the desired cysteine residues was performed using the QuickChange Lightning Mutagenesis Kit (Agilent) following the manufacturer’s protocol. Expression cultures were grown to an OD_600_ of 0.8–1 and induced with 0.6 mM IPTG, and expression was performed for 5 h at 37°C. For NMR experiments, the c-Myc constructs were uniformly labeled with ^15^N/^13^C isotopes by growing the bacterial cultures in minimal media supplemented with ^15^NH_4_Cl and ^13^C-glucose. Cells were harvested by centrifugation at 5000 g for 15 min, and pellets were stored at −80°C until use.

Cell pellets were resuspended in native lysis buffer (100 mM NaH_2_PO_4_ (sodium dihydrogen phosphate), 10  mM Tris-HCl, 300  mM NaCl, and 1 mM TCEP, pH 8), sonicated on ice at 50% amplitude (8 × 15 s) using a Qsonica sonicator, and centrifuged at 15,000 g for 45  min. The pellet was resuspended in denaturing lysis buffer (100 mM NaH_2_PO_4_, 10  mM Tris-HCl, 8 M urea, 1 mM TCEP, pH 8), followed by a second round of sonication and centrifugation. The supernatant was loaded onto a HisTrap HP Ni column equilibrated with denaturing lysis buffer and washed with wash buffer (100 mM NaH_2_PO_4_, 10  mM Tris-HCl, 8 M urea, and 1 mM TCEP, pH 8). The protein was eluted using elution buffer (100 mM NaH_2_PO_4_, 10  mM Tris-HCl, 1 mM TCEP, and 8 M urea, pH 4.5). Eluted protein was dialyzed overnight against dialysis buffer (50  mM NaH_2_PO_4_, 10  mM Tris-HCl, 100  mM NaCl, 5% glycerol, 1 mM TCEP, 5 mM EDTA, pH 8) at 4°C. The c-Myc protein was further purified by size exclusion chromatography (HiLoad 16/600 Superdex 75 , Cytiva) in gel filtration buffer (20  mM HEPES, pH 6.9, 300  mM NaCl, 1  mM TCEP, 5% glycerol).

The kinase domain of AurA (residues 122–403) with an N-terminal cleavable His-tag was expressed in BL21(DE3)-RIL cells O/N at 18°C and purified as described previously [39]. We used a cysteine-lite form of AurA containing C290S and C393S mutations, and an L225C mutation for fluorophore incorporation.

### Labeling of c-Myc^1-331^ constructs and AurA

c-Myc^1-331^ and AurA were purified in 20 mM HEPES pH 7.4, 150 mM NaCl, 5% (v/v) glycerol, 5 mM DTT or 50 mM HEPES pH 7.5, 300 mM NaCl, 10% (v/v) glycerol, and 1 mM DTT, respectively. Each was desalted into identical buffers lacking DTT, and immediately mixed with 2× Alexa Fluor 488 C5 Maleimide (c-Myc^1-331^) (Thermo Fisher) or DABCYL-Plus C2 maleimide (AurA) (AnaSpec). Labeling reactions were gently rocked at room temperature for 1 h (c-Myc^1-331^) and 2 h (AurA). Excess label was removed after incubation by desalting, and labeling efficiency was confirmed through intact protein LC/MS (Supplementary Figure S3a,b,d,e).

### *In vitro* phosphorylation of c-Myc

Double phosphorylation of Alexa 488-labeled c-Myc^1-331^ was performed at 20–40 mM c-Myc^1-331^ in phosphorylation buffer (20 mM HEPES pH 7.0, 100 mM NaCl, 50 mM MgCl_2_, 5% (v/v) glycerol). 10 nM T160-phosphorylated CDK2, 30 nM Cyclin A, 50 nM GSK3b, and 500 mM ATP were mixed with c-Myc^1-331^ in phosphorylation buffer and incubated at room temperature for 1 h with gentle rocking. Excess ATP was removed by desalting the reaction into 20 mM HEPES pH 7.5, 150 mM NaCl, 5% (v/v) glycerol. Phosphorylation was confirmed with Phos-Tag gels and intact protein and tandem mass spectrometry (Supplementary Figure S3a-c).

### Time-resolved fluorescence

Intermolecular FRET was measured using a custom fluorescence lifetime plate reader (Photonic Pharma) [29], using doubly phosphorylated c-Myc^1-331^ protein samples individually labeled at one of eight natively occurring cysteine residues (C25S, 70, 117, 133, 171, 188, 208, 300). Experiments were performed in 384-well black bottom plates (Costar). Alexa 488 labeled c-Myc^1-331^ was used at a constant concentration of 50 nM in all experiments. Serially diluted DABCYL-labeled AurA and unlabeled AurA (donor-only controls) were mixed with c-Myc^1-331^ to final AurA concentrations spanning 0–12.8 μM. Plates were incubated at room temperature for at least 30 min to reach equilibrium before initiating fluorescence lifetime measurements. Lifetime values were determined by a fitting procedure in MATLAB and Python in which individual fluorescence decays were modeled as a convolution of an exponential decay with the experimentally determined instrument response function. Lifetime values were plotted in GraphPad Prism and used to calculate average FRET efficiency and *Kd* values.

### Steady-state fluorescence anisotropy

Fluorescence anisotropy was measured on a Tecan Infinite M1000 PRO Microplate Reader, using the same experimental plates created for the time-resolved fluorescence measurements. Fluorescence anisotropy was measured at an excitation wavelength of 470 nm and an emission wavelength of 516 nm, both with bandwidths of 5 nm. The protocol took 50 reads and calculated the *z*-position from the control well used to generate an optimal gain value. Optimal gains were consistent between all plates measured with minimal variation. Fluorescence anisotropy values were calculated from parallel and perpendicular emission intensity values using the formula:


$$\text{Anisotropy}=\frac{I^{parallel}-I^{perpendicular}}{I^{parallel}+2I^{perpendicular}}$$

Anisotropy values were fit to a 1:1 binding model in GraphPad Prism to calculate *Kd* values.

### NMR spectroscopy

For all NMR experiments, ^15^N- and/or ^13^C-labeled c-Myc samples were prepared in buffer (20  mM HEPES, pH 6.9, 300  mM NaCl, 1  mM TCEP, 5% glycerol) in 90% H_2_O and 10% D_2_O. All NMR experiments were recorded on a Bruker 900 MHz spectrometer equipped with cryoprobe. Triple resonance experiments (HNCA, HN(CA)CO, HNCACB, HNCO, and CBCACONH) were conducted with a protein concentration of ~450 μM at 303K. Spectra were recorded with a sweep width of 16 ppm in the ^1^H dimension and 36 ppm in the ^15^N dimension, with offset values set to 4.7 and 120 ppm, respectively. NMR spectra were processed using TOPSPIN 3.2 and analyzed with CARA software. Integrity of NMR samples was verified by acquiring matched HSQC spectra before and after triple resonance experiments.

### Phosphorylation of NMR samples

*In vitro* double phosphorylation of c-Myc^1-88^ was performed using phosphorylation buffer containing 20 mM HEPES pH 7.0, 100 mM NaCl, 50 mM MgCl 2, and 5% (v/v) glycerol. Briefly, 20 µM c-Myc^1-88^ and 10 nM phosphorylated CDK2, 30 nM Cyclin A, 50 nM GSK3b, and 500 μM ATP were mixed in phosphorylation buffer and incubated at room temperature for 1 h with gentle rocking. Excess ATP was removed by desalting the reaction into 20 mM HEPES pH 7.5, 150 mM NaCl, 5% (v/v) glycerol. Phosphorylation was confirmed with Phos-Tag gels and intact mass spectrometry and monitored by NMR HSQC spectroscopy.

### Mass spectrometry

Intact protein mass spectrometry was performed on an Orbitrap Elite (ThermoFisher). Samples were desalted into 10 mM ammonium acetate and concentrated to 50 ml of 20 mM protein. After washing the column and lines of the instrument, 5 ml of each sample was loaded at a flow rate of 200 ml per min. The c-Myc samples were run before the AurA samples, and all of the samples eluted 3 min post-injection. Data were analyzed on Protein Deconvolution software from Thermo Fisher. The isotopically unresolved analysis method was used with an m/z range of 500–2000 and a mass tolerance of 0.05. For the c-Myc samples, the target mass was set to 36,827 Da and the output mass range was set to 30,000–45,000 Da. For the AurA sample, the target mass was set to 33,488 Da for the cysteine-lite construct and 32,934 Da for the wildtype kinase domain construct. The output data on the most abundant species were discretized by the program and plotted in GraphPad Prism to adjust the axes to the mass area of interest and obtain high-resolution plots for inclusion in the manuscript.

Tandem LC-MS/MS was performed on an Orbitrap Tribrid Eclipse (ThermoFisher). Samples were concentrated to about 0.1 mg/ml in 20 ml of 50 mM Tris-HCl pH 7.8 and were prepared with an in-solution trypsin protein digest. Known sequences of the c-Myc constructs were used to perform a targeted phosphoproteomics analysis in PEAKS software. The peptide fragment spectra were analyzed to confirm phosphorylation sites identified by the PEAKS software.

### HSQC titration experiments

Two-dimensional ^15^N-^1^H HSQC titration experiments were conducted by incrementally adding unlabeled AurA to deuterated and ^15^N-labeled c-Myc. The c-Myc concentration was kept constant, while the AurA concentration was increased in 0.25 equivalent steps until reaching a molar ratio of 1:1. Addition of AurA led to substantial loss in signal as observed in both 1D ^1^H NMR spectra (Supplementary Figure S9a,b) and ^15^N-^1^H HSQC. CSPs were calculated for each titration point to identify and characterize the binding regions on c-Myc (Supplementary Figure S5 and S8). ^1^H-^15^N HSQC titrations were recorded at a c-Myc concentration of 100 μM. CSPs were calculated using the equation:


$$\Delta \delta =\sqrt{{\left(\Delta \delta H\right)}^{2}+{\left(\Delta \delta N/5\right)}^{2}}$$

where ΔδH and ΔδN are the changes in chemical shifts of proton and nitrogen, respectively.

### AlphaFold modeling of c-Myc and AurA kinase interaction

For modeling of the c-Myc^1-331^ and AurA^122-403^ interaction in their unphosphorylated states, we utilized the AlphaFold 3 Server for protein structure prediction, as described by Abramson et al. [33]. Three independent prediction runs were performed, each generating five relaxed models of the c-Myc/AurA complex, resulting in a total of 15 models. Inspection of the predicted alignment error plots for each model identified 12 models in which a portion of MBI was ordered in a defined orientation with respect to the kinase domain, and manual inspection of the models confirmed the formation of the same interaction in each of these models.

## Supplementary material

online supplementary figures

## Data Availability

The data that support the findings in this study are available within the paper (main and supplementary sections).

## Abbreviations

AurA: Aurora A
CSP: chemical shift perturbation
FRET: Förster resonance energy transfer
MB: Myc box
