# Supplementary material for: Aurora A binds to the transactivation domain of c-Myc and recognizes the phosphorylated N-terminal degron motif
Source: Biochem J. 2025 Apr 16;482(8):369–81. doi: 10.1042/BCJ20240726 (PMC12203944; doi:10.1042/BCJ20240726)
Supplement: online supplementary figures [file BCJ-482-08-BCJ20240726-s001.docx]

**SUPPLEMENTARY FIGURES**

**Aurora A binds to the transactivation domain of c-Myc and recognizes the phosphorylated N-terminal degron motif**

Nidhi Joshi^1^, Katie M. Dunleavy^1^, Kaitlin M. Beel^1^, Tiffany A. Engel^1^, Andrew R. Thompson^2^, Felix L. John^2^, David D. Thomas^2^ and Nicholas M. Levinson^1*^

^1^Department of Pharmacology and Masonic Cancer Center, University of Minnesota, 312 Church St. SE., Minneapolis, MN 55455

^2^Department of Biochemistry, Molecular Biology, and Biophysics, University of Minnesota, 312 Church St. SE, Minneapolis, MN 55455

*To whom correspondence should be addressed ([nml@umn.edu](mailto:nml@umn.edu))

**
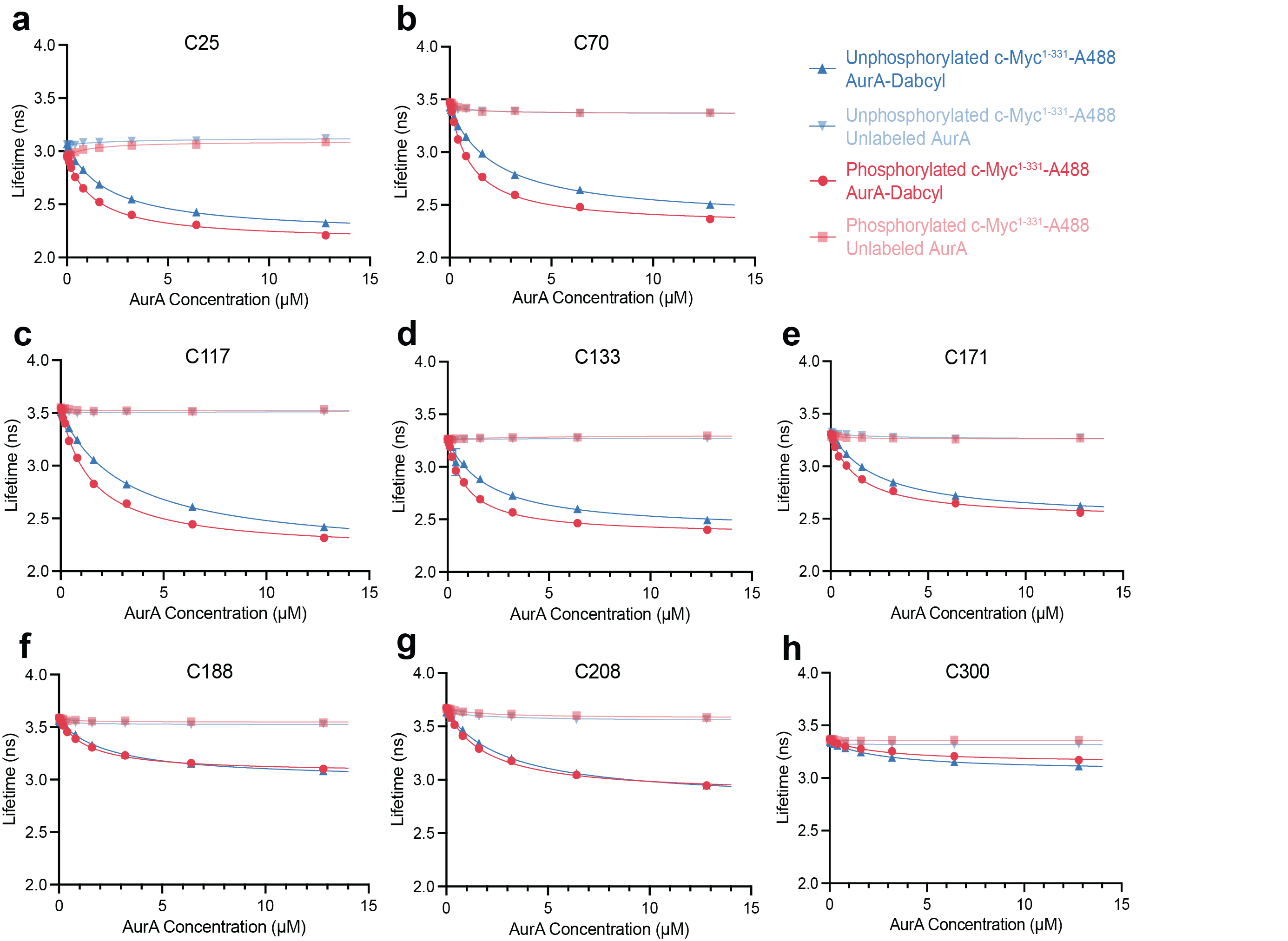
**

**Figure S1. Fluorescence lifetime measurements for AurA titrations with unphosphorylated and phosphorylated c-Myc^1-331^.** Fluorescence lifetimes measured over an AurA titration for unphosphorylated (blue) and phosphorylated (red) c-Myc^1-331^ labeled with A488 on **a**) C25, **b**) C70, **c**) C117, **d**) C133, **e**) C171, **f**) C188, **g**) C208, and **h**) C300. Dark curves show a titration of dabcyl-labeled AurA, and faded curves show a titration of unlabeled AurA (donor-only control). Data represent mean ± s.d.; n=3. A single representative example from three independent experiments is shown.

**
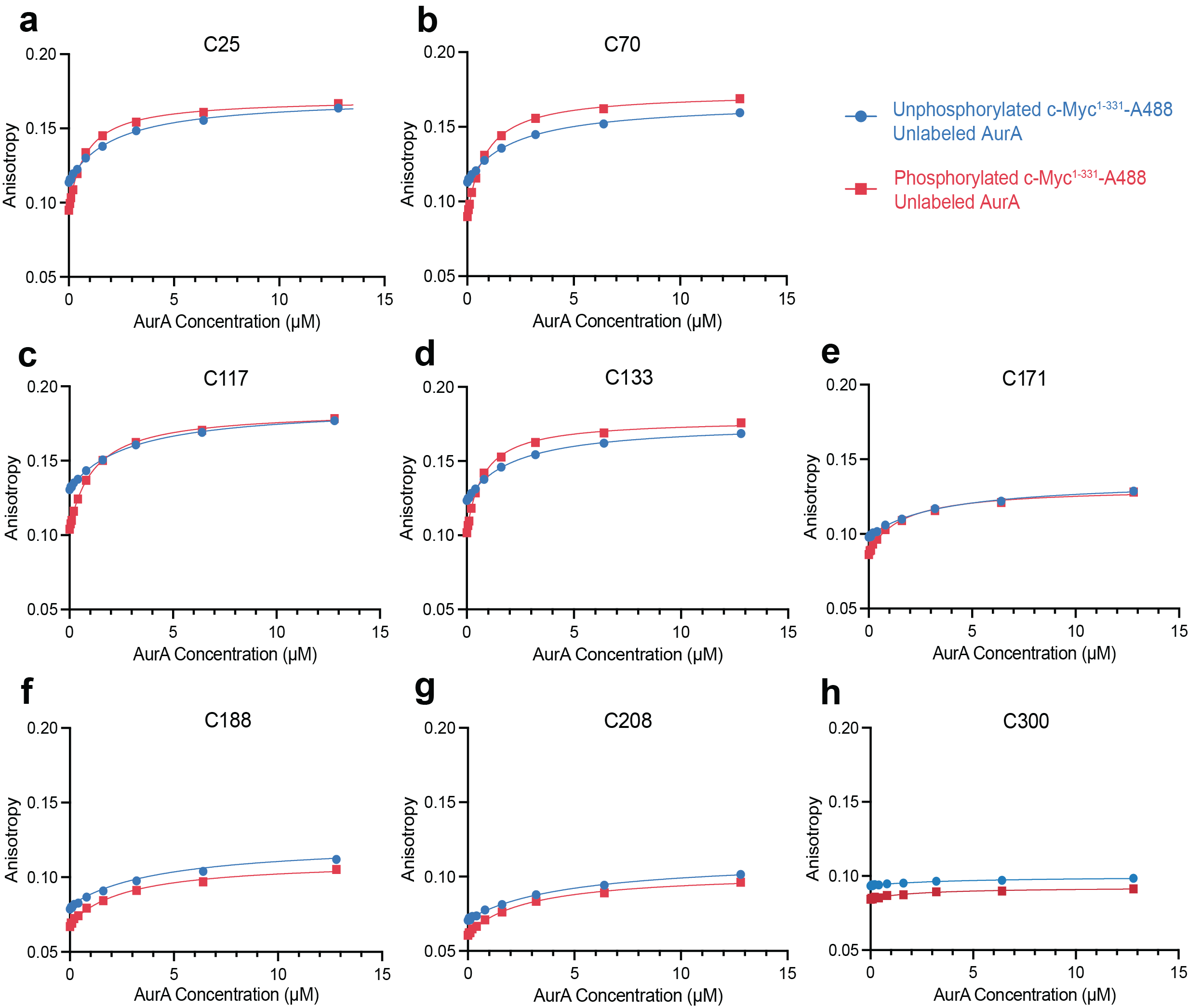
**

**Figure S2. Fluorescence anisotropy measurements for AurA titrations with unphosphorylated and phosphorylated c-Myc^1-331^.** Fluorescence anisotropy measured over a titration of unlabeled AurA for unphosphorylated (blue) and phosphorylated (red) c-Myc^1-331^ labeled with A488 on **a**) C25, **b**) C70, **c**) C117, **d**) C133, **e**) C171, **f**) C188, **g**) C208, and **h**) C300. Data represent mean ± s.d.; n=3. A single representative example from three independent experiments is shown.


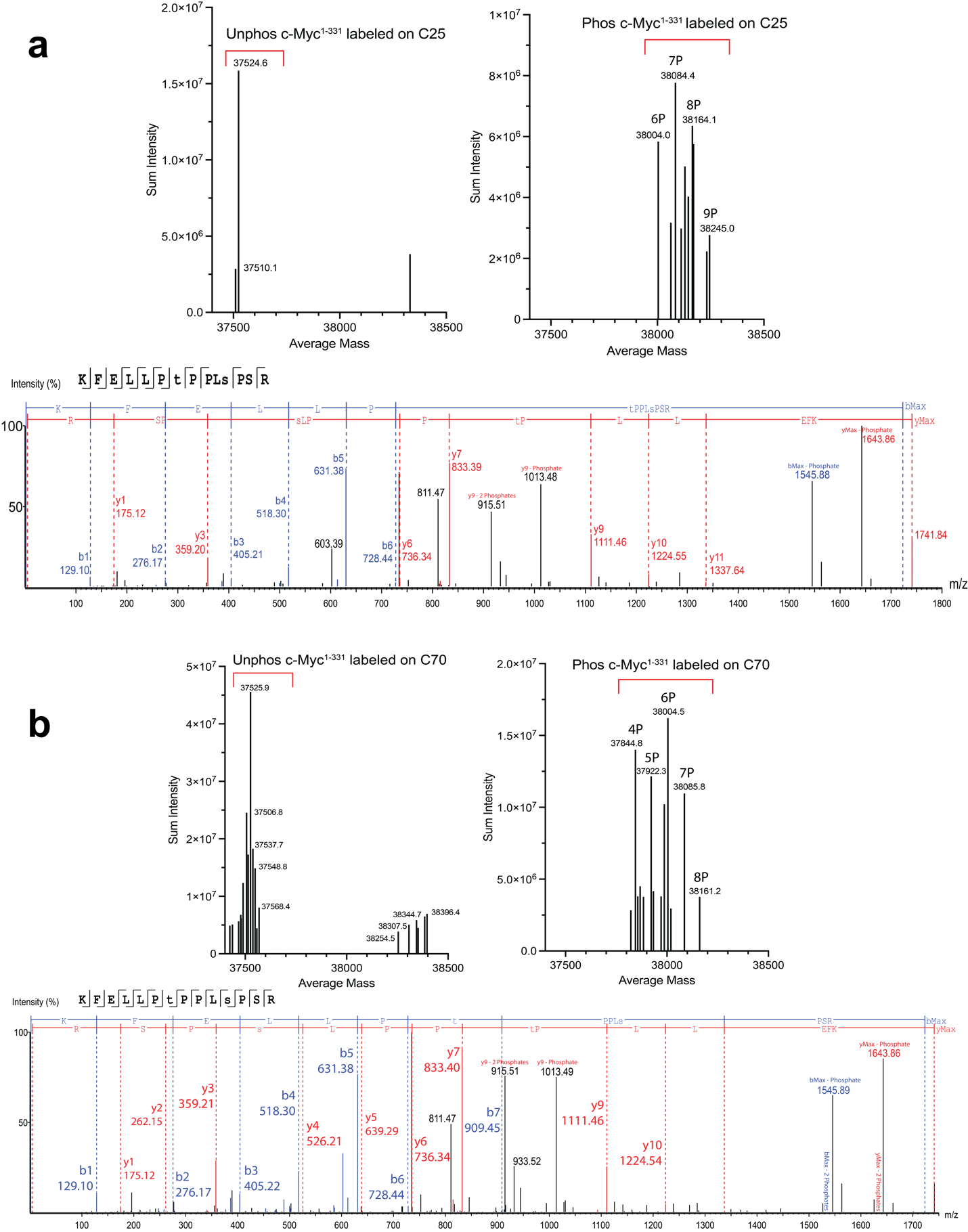


**
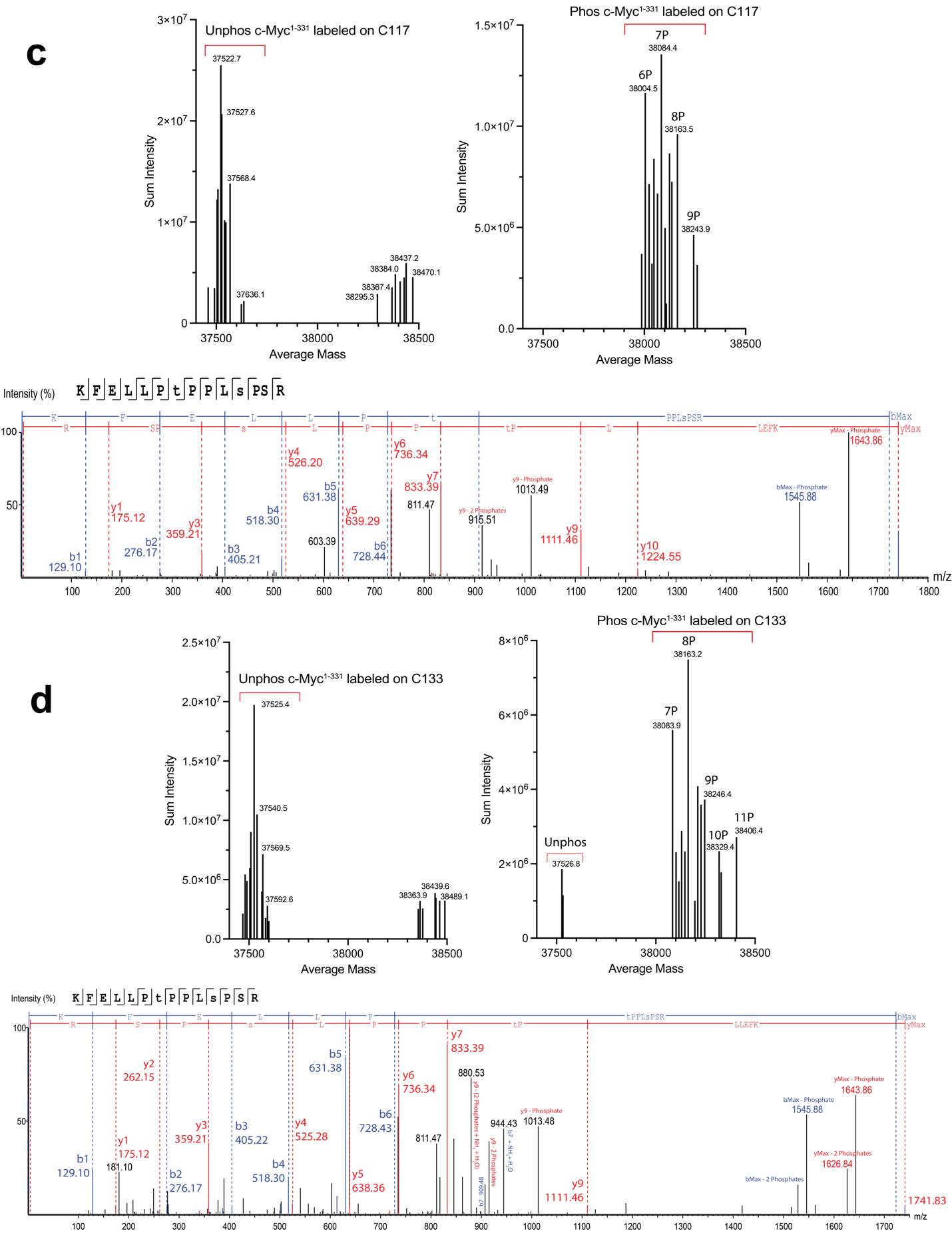
**

**
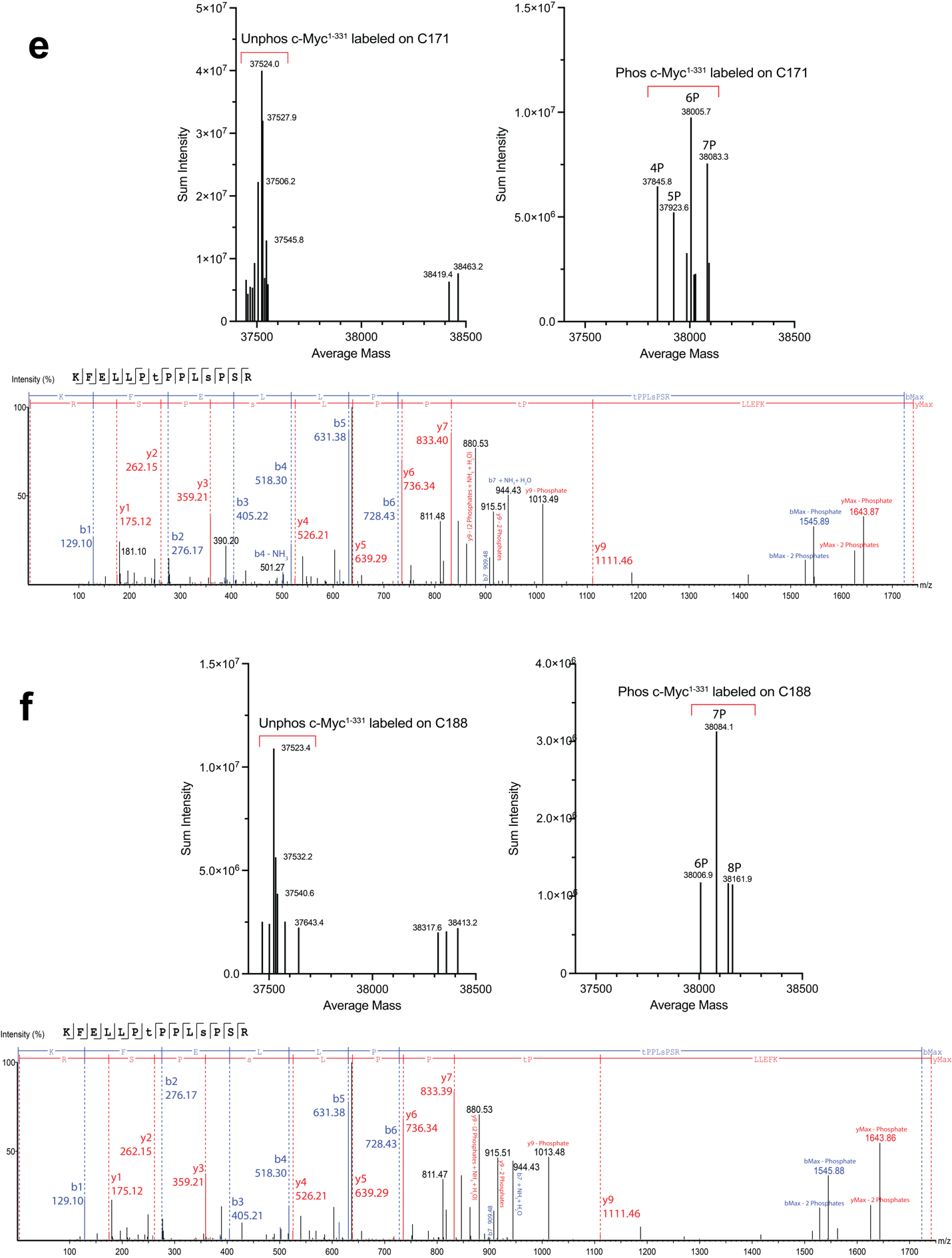
**

**
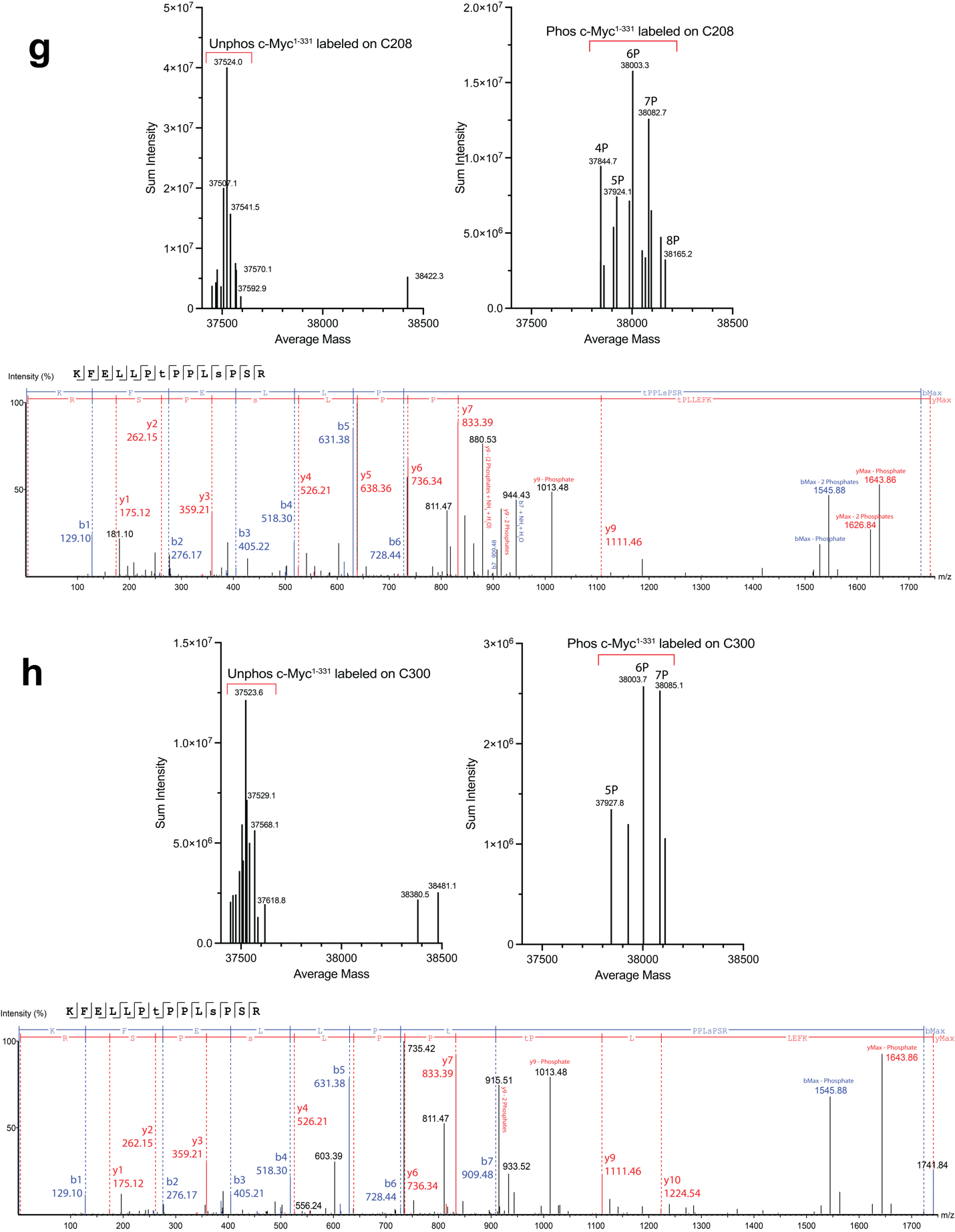
**

**
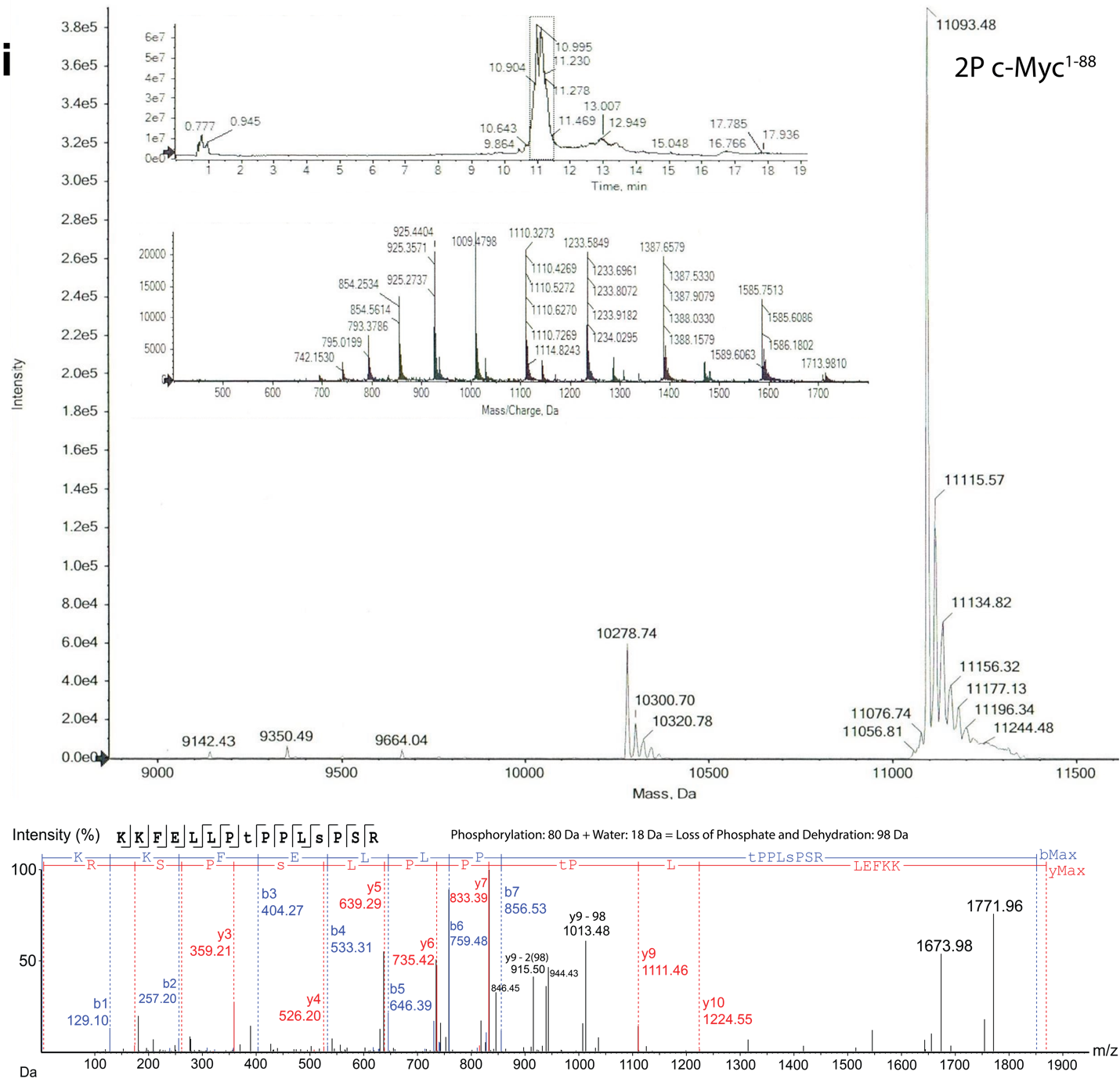
**

**
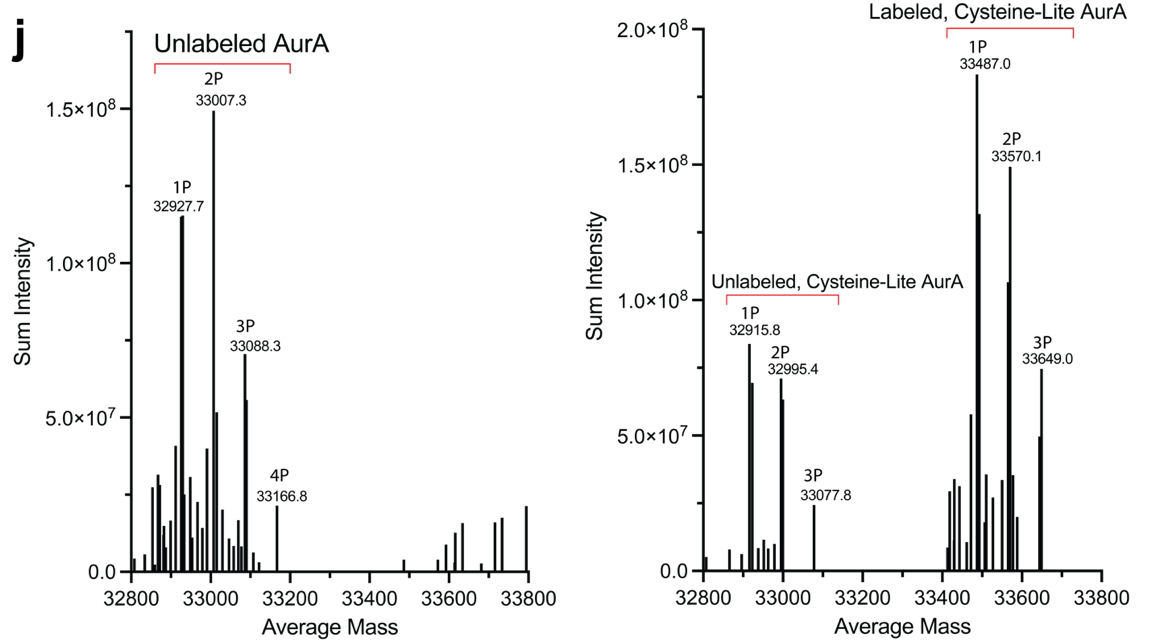
**

**Figure S3. Mass spectrometry data on c-Myc and AurA samples used in fluorescence experiments**. **a**) Intact mass spectrometry data on unphosphorylated (top left) and phosphorylated (top right) c-Myc^1-331^ labeled with Alexa 488 C5 maleimide (A488) on C25. Tandem LC-MS/MS confirmed the phosphorylation of T58 and S62 from the y6 and y9 ions (bottom). **b)** Intact mass spectrometry data on unphosphorylated (top left) and phosphorylated (top right) c-Myc^1-331^ labeled with A488 on C70**.** Tandem LC-MS/MS confirmed the phosphorylation of T58 and S62 from the y4, b7, and y9 ions (bottom). **c)** Intact mass spectrometry data on unphosphorylated (top left) and phosphorylated (top right) c-Myc^1-331^ labeled with Alexa 488 C5 maleimide (A488) on C117. Tandem LC-MS/MS confirmed the phosphorylation of T58 and S62 from the y4 and y9 ions (bottom). **d)** Intact mass spectrometry data on unphosphorylated (top left) and phosphorylated (top right) c-Myc^1-331^ labeled with A488 on C133**.** Tandem LC-MS/MS confirmed the phosphorylation of T58 and S62 from the y9 and y4 ions (bottom). **e)** Intact mass spectrometry data unphosphorylated (top left) and phosphorylated (top right) c-Myc^1-331^ labeled with A488 on C171**.** Tandem LC-MS/MS confirmed the phosphorylation of T58 and S62 from the y9 and y4 ions (bottom). **f)** Intact mass spectrometry data on unphosphorylated (top left) and phosphorylated (top right) c-Myc^1-331^ labeled with A488 on C188**.** Tandem LC-MS/MS confirmed the phosphorylation of T58 and S62 from the y9 and y4 ions (bottom). **g)** Intact mass spectrometry data on unphosphorylated (top left) and phosphorylated (top right) c-Myc^1-331^ labeled with A488 on C208**.** Tandem LC-MS/MS confirmed the phosphorylation of T58 and S62 from the y9 and y4 ions (bottom). **h)** Intact mass spectrometry data on unphosphorylated (top left) and phosphorylated (top right) c-Myc^1-331^ labeled with A488 on C300**.** Tandem LC-MS/MS confirmed the phosphorylation of T58 and S62 from the y9 and y4 ions (bottom). **i)** Intact mass spectrometry data on doubly phosphorylated c-Myc^1-88^ (top). Tandem LC-MS/MS confirms the phosphorylation of T58 and S62 from the y9 and y4 ions (bottom). **j)** Intact mass spectrometry on unlabeled and labeled AurA kinase. Unlabeled AurA containing C290S and C393S mutations that was used for donor-only controls in fluorescence experiments (left). Dabcyl-labeled AurA containing C290S, C393S, and L225C mutations was used in donor-acceptor fluorescence experiments (right).


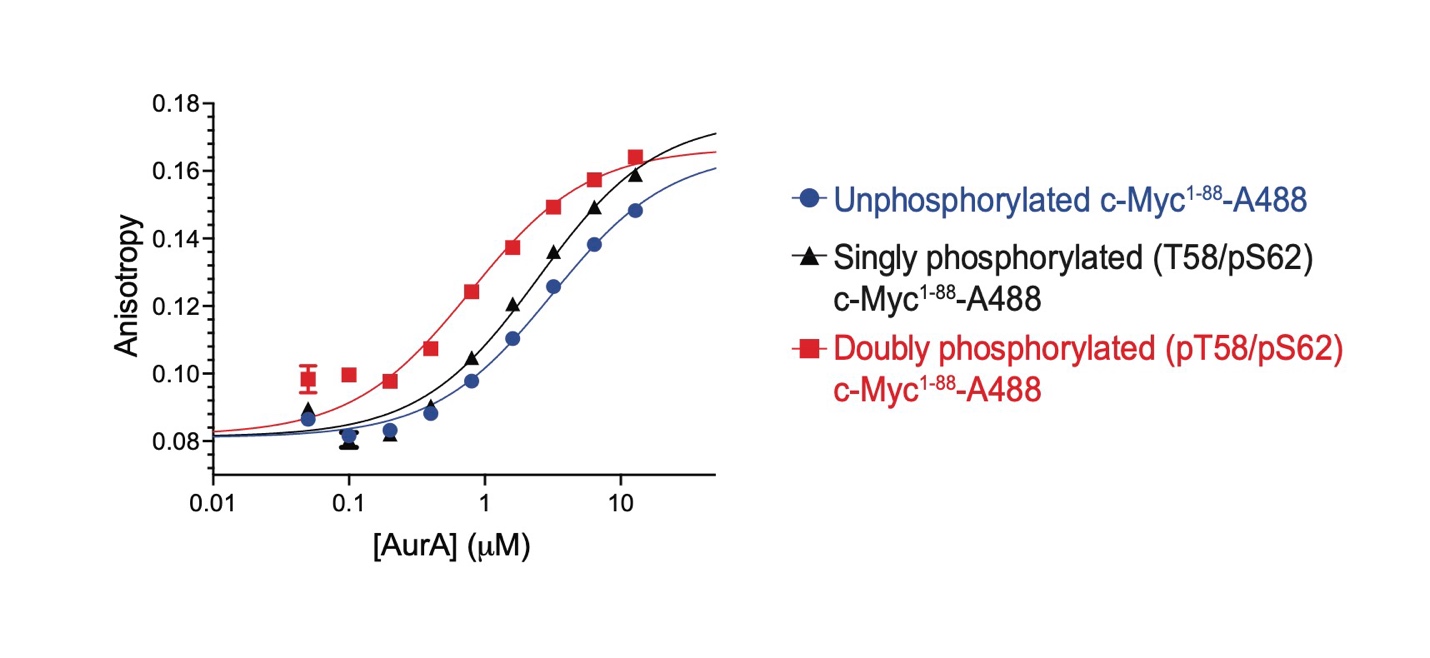


**Figure S4. Fluorescence anisotropy measurements for AurA titrations with unphosphorylated, singly (T58/pS62)- and doubly (pT58/pS62)-phosphorylated c-Myc^1-88^.** Fluorescence anisotropy measured for a titration of unlabeled AurA with unphosphorylated (blue) and singly- (black) and doubly-phosphorylated (red) c-Myc^1-88^ labeled with A488 on C70.

**
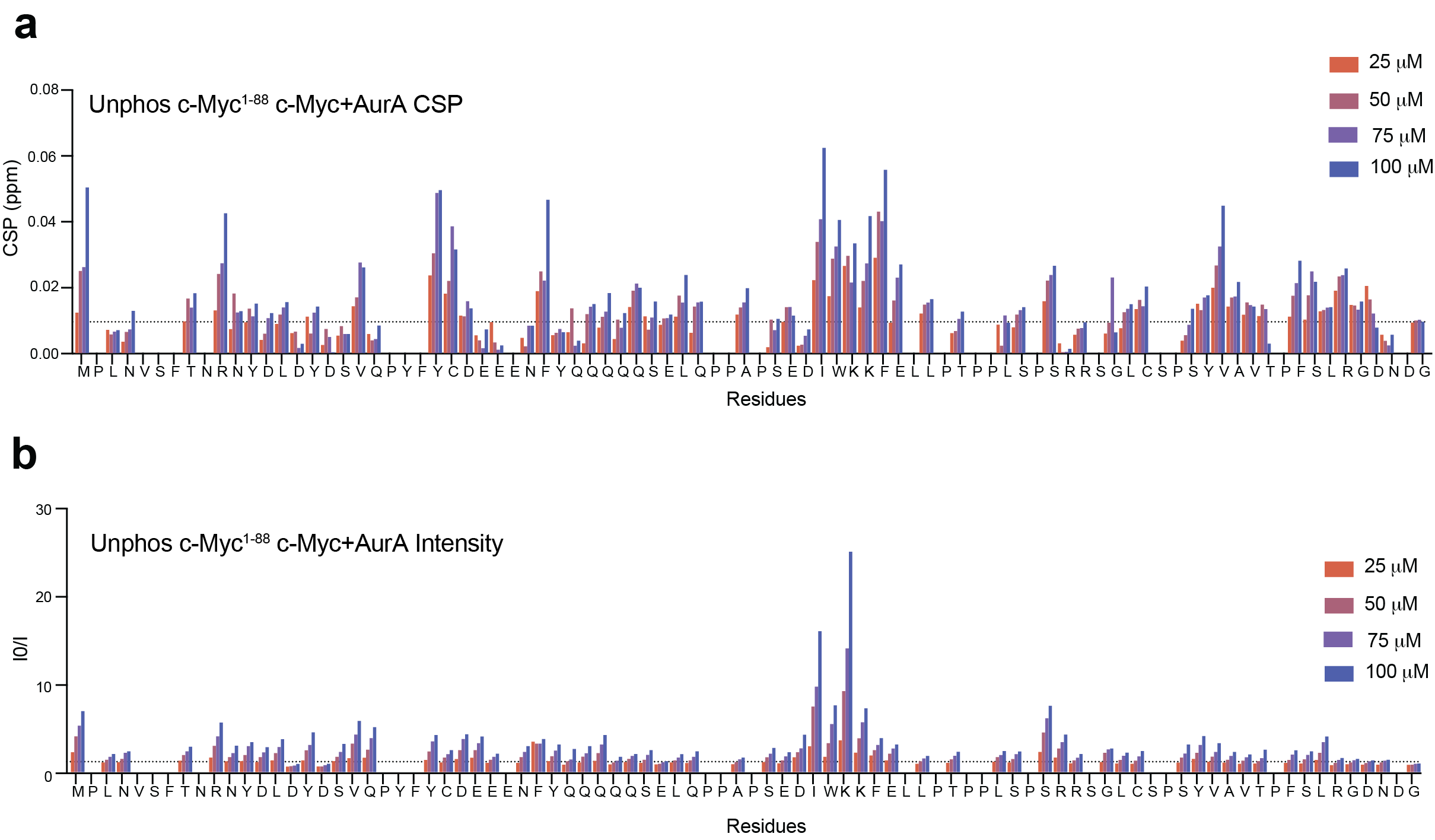
**

**Figure S5. CSP and intensity change analysis for unphosphorylated c-Myc^1-88^-AurA interactions.** **a**) Chemical shift perturbations (CSPs) and **b**) intensity reduction profiles (I0/I) of unphosphorylated c-Myc^1-88^ observed with increasing concentrations of AurA (25 µM, orange, 50 µM, light purple, 75 µM, dark purple,100 µM, blue).

­
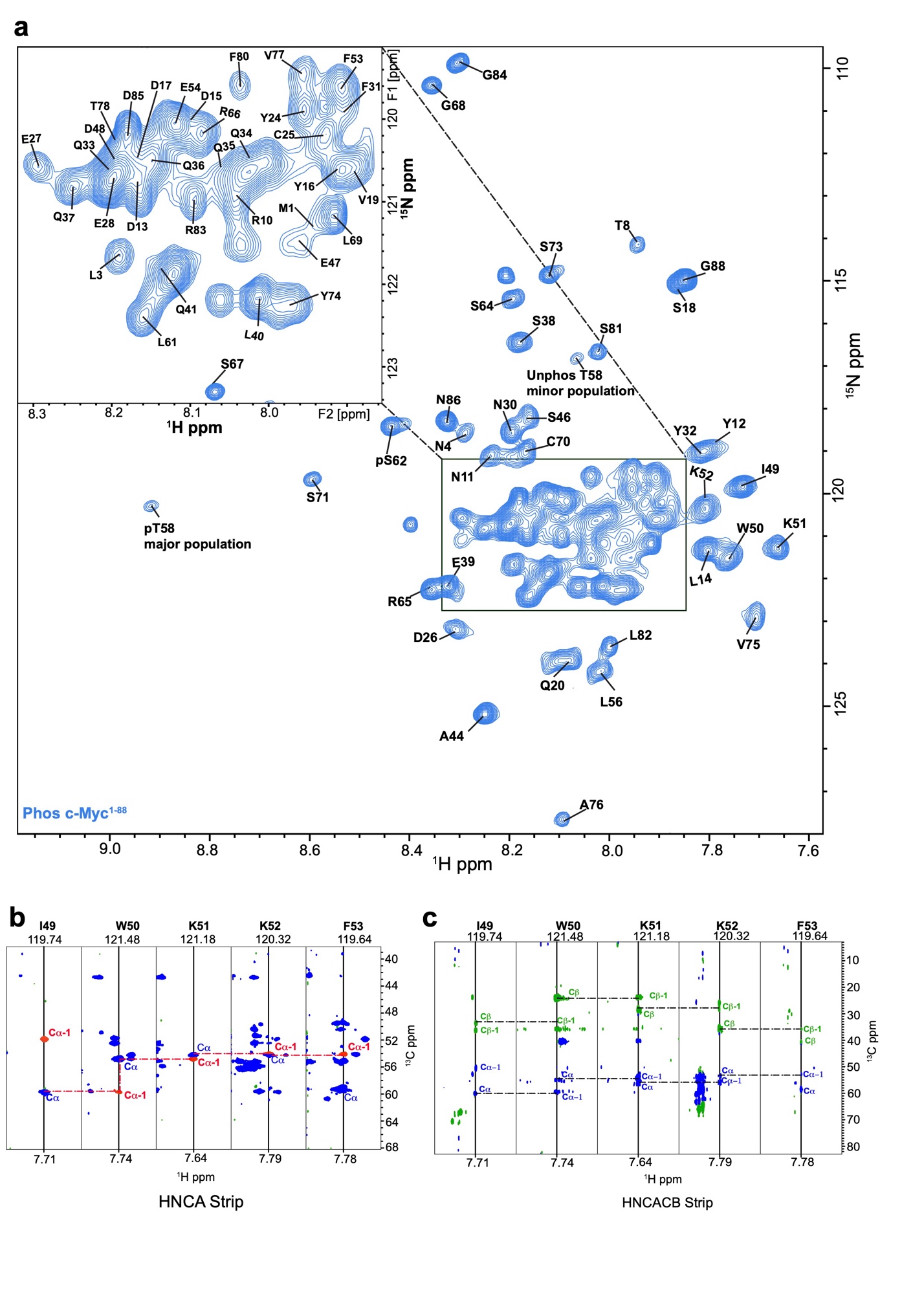


**Figure S6.** **NMR** **assignment of doubly-phosphorylated c-Myc^1-88^. a**) Two-dimensional [^1^H, ^15^N]-HSQC spectrum of doubly-phosphorylated c-Myc^1-88^, showing backbone resonance assignments for 69 of 77 non-proline residues. The inset highlights an enlarged view of the central region for improved clarity. **b**) HNCA and **c**) HNCACB strip plots illustrating sequential connectivities for the stretch of residues I49 to F53.


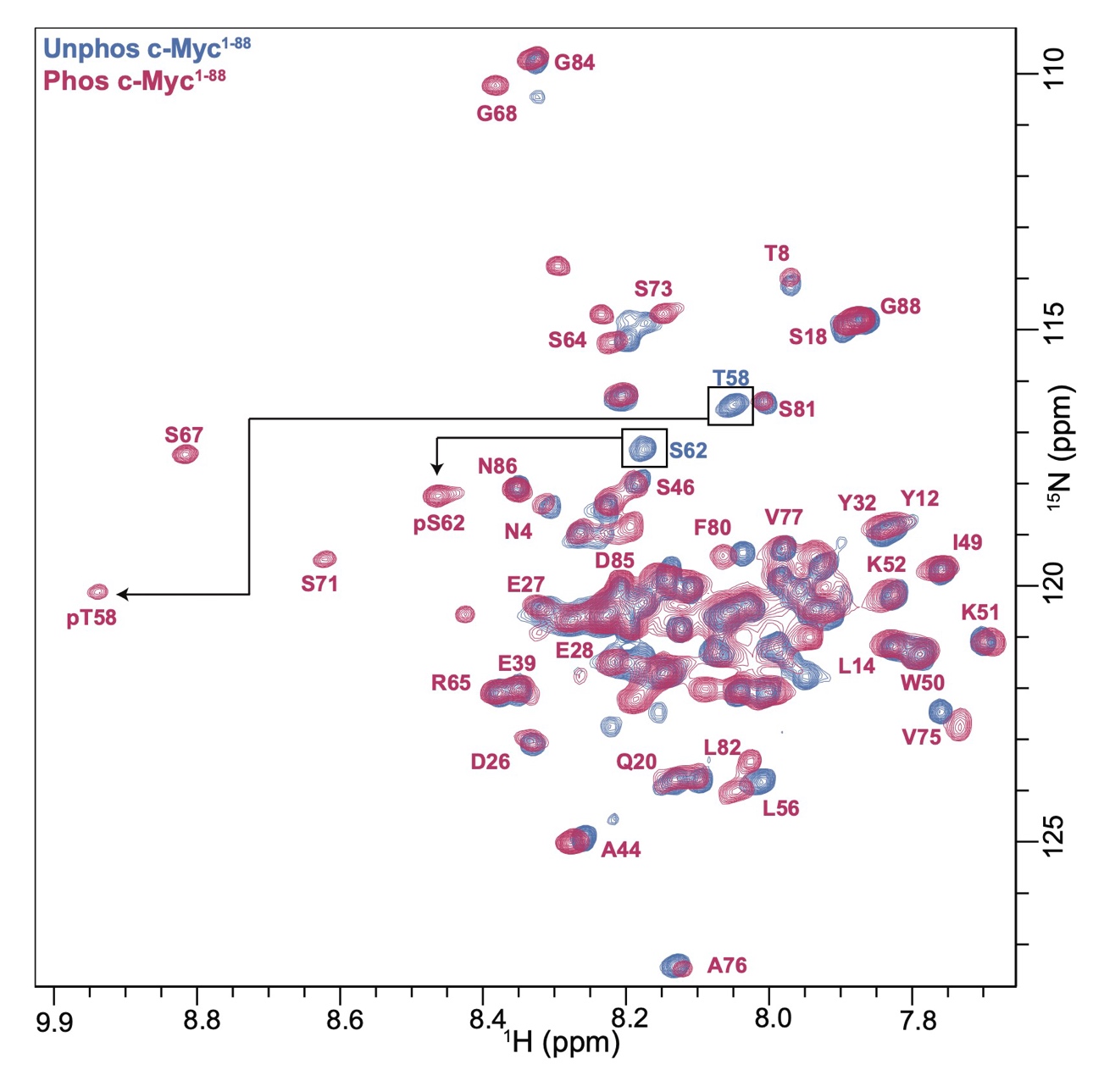


**Figure S7. Overlay of [^1^H, ^15^N]-HSQC spectra for unphosphorylated and double-phosphorylated c-Myc^1-88^**. A comparison of unphosphorylated c-Myc (blue) and doubly phosphorylated c-Myc (magenta) is shown. The substantial downfield shifts observed for T58 and S62 upon phosphorylation are highlighted.

**
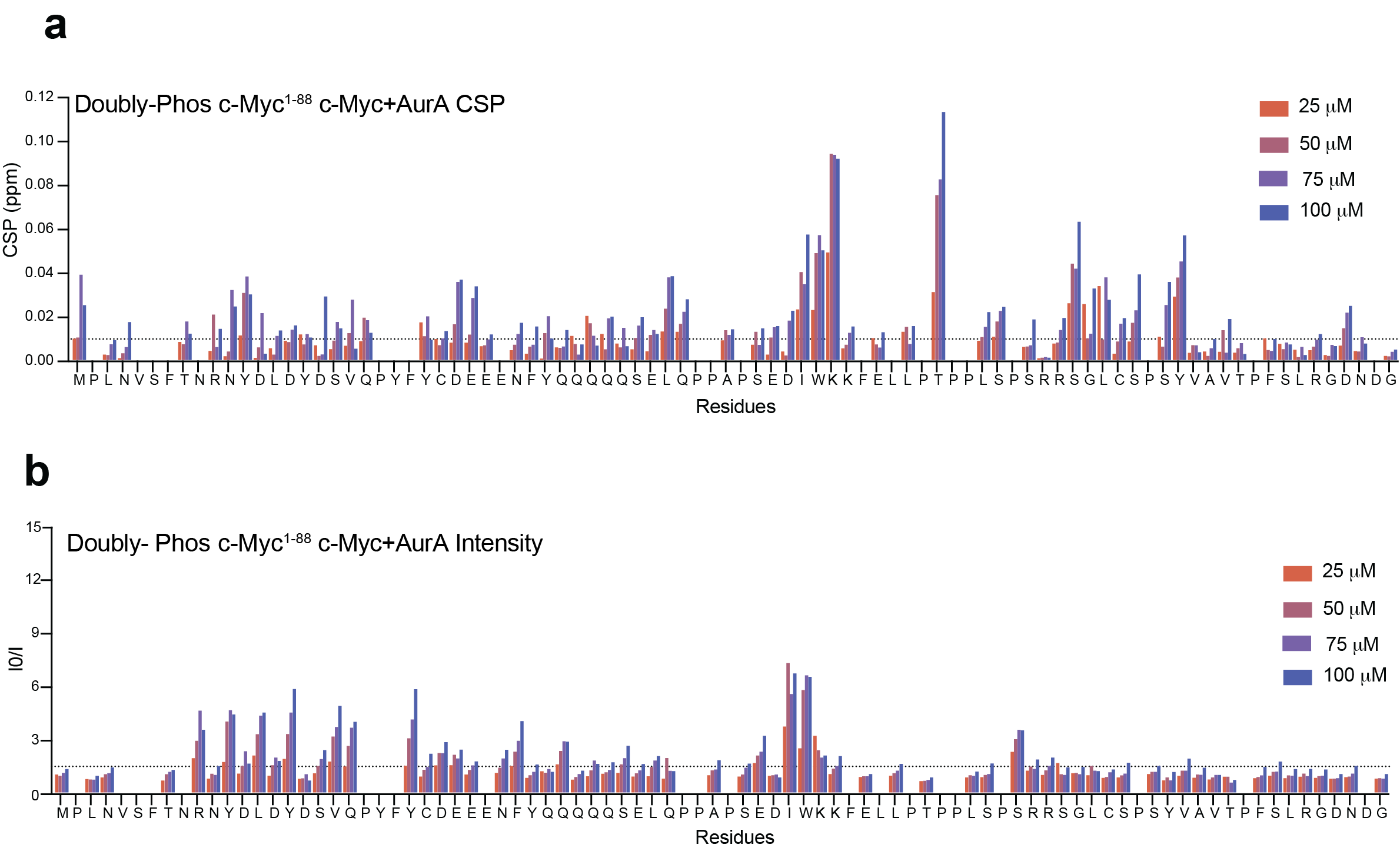
**

**Figure S8. CSP and intensity change analysis for doubly-phosphorylated c-Myc^1-88^-AurA interactions.** **a**) Chemical shift perturbations (CSPs) and **b**) intensity reduction profiles of doubly-phosphorylated c-Myc^1-88^ upon titration with increasing concentrations of AurA (25 µM, orange, 50 µM, light purple, 75 µM, dark purple,100 µM, blue).


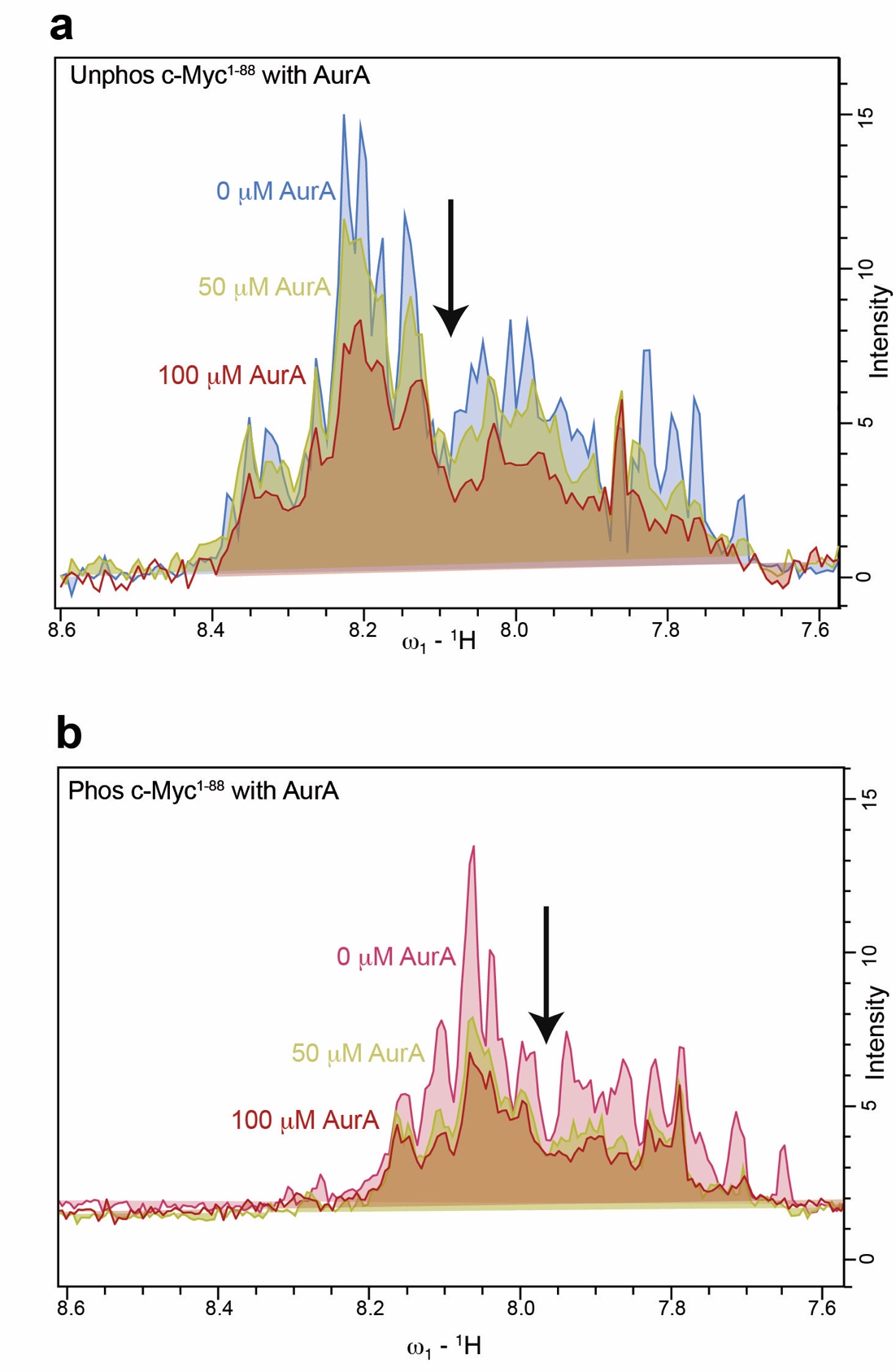


**Figure S9.** **Overlay of 1D ^1^H NMR spectra of unphosphorylated and doubly-phosphorylated c-Myc^1-88^-AurA interactions. a)** Overlay for the unphosphorylated c-Myc^1-88^-AurA interaction at AurA concentrations of 0 µM (blue), 50 µM (pale yellow), and 100 µM (red). **b)** Overlay for the doubly-phosphorylated c-Myc^1-88-^AurA interaction at AurA concentrations of 0 µM (magenta), 50 µM (pale yellow), and 100 µM (red).

**Figure S10.** **Overlay of [^1^H, ^15^N]-HSQC spectra of unphosphorylated c-Myc^1-88^ with AurA.** **a)** Overlay of [^1^H, ^15^N]-HSQC spectra of unphosphorylated c-Myc^1-88^ (blue) with increasing concentrations of AurA (25 µM to 100 µM). **b)** Enlarged view of representative peaks from MB0 and MBI regions (R10, K51, S64, R65, F80, and R83) showing signal loss and/or chemical shift changes. Colors represent AurA concentrations: 25 µM (green), 50 µM (yellow), 75 µM (orange), and 100 µM (red).


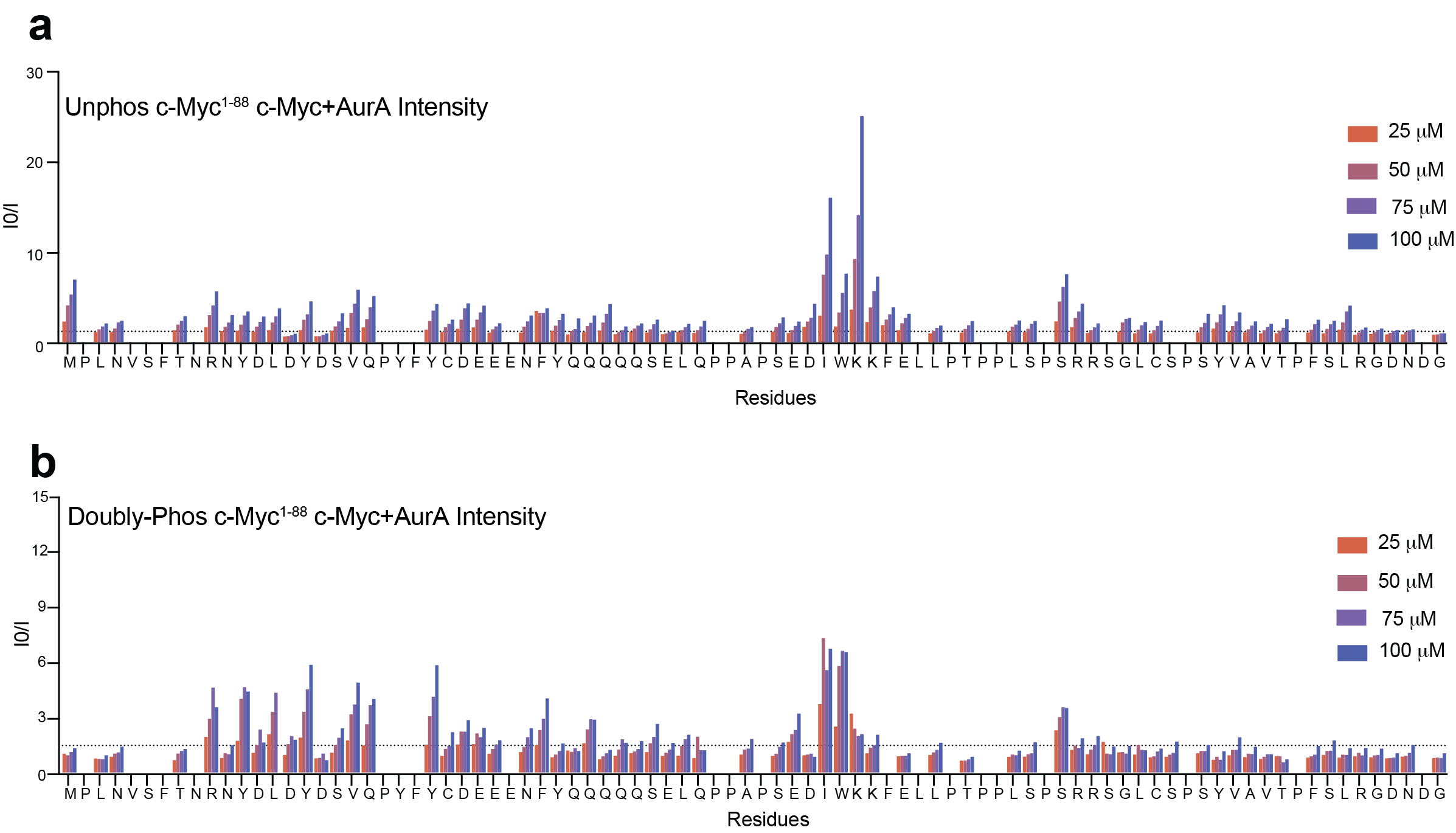


**Figure S11. Comparison of Fold Intensity Changes for Unphosphorylated and Doubly-Phosphorylated c-Myc ^1-88^. a**) Fold intensity changes of unphosphorylated c-Myc ^1-88^ residues upon titration with AurA at increasing concentrations (25 µM, orange, 50 µM, light purple, 75 µM, dark purple,100 µM, blue). Values >1 indicate signal loss. The dashed line shows the average I₀/I at 25 µM AurA. **b**) Fold intensity changes of doubly-phosphorylated c-Myc ^1-88^ residues upon titration with AurA (25 µM, orange, 50 µM, light purple, 75 µM, dark purple,100 µM, blue). Values >1 indicate signal loss. The dashed line shows the average I₀/I at 25 µM AurA.

**Figure S12.** **Overlay of [^1^H, ^15^N]-HSQC spectra of doubly-phosphorylated c-Myc^1-88^ with AurA.** **a)** Overlay of [^1^H, ^15^N]-HSQC spectra of doubly-phosphorylated c-Myc^1-88^ (magenta) with increasing concentrations of AurA (25 µM to 100 µM). **b)** Enlarged view of representative peaks from MB0 and MBI regions (Y12, Y32, K51, and S67) showing signal loss and/or chemical shift changes. Colors represent AurA concentrations: 25 µM (green), 50 µM (yellow), 75 µM (orange), and 100 µM (red).


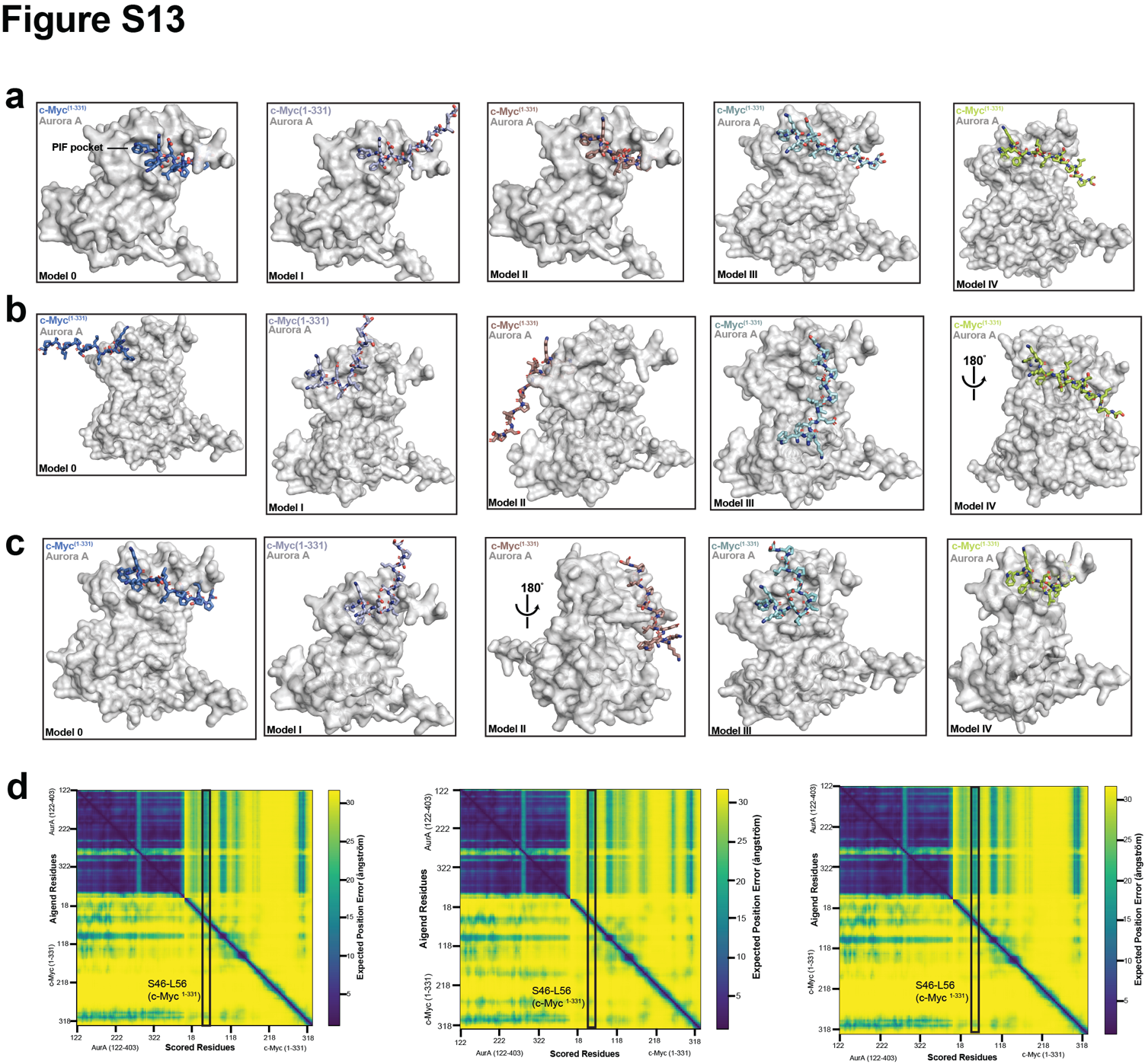


**Figure S13.** **AlphaFold Models of the c-Myc-AurA Complex.**

**a-c)** All predicted models of the c-Myc-AurA complex from three independent AlphaFold runs. Each panel displays five models from a single run (5 per run, 15 total). PIF pocket is highlighted in first panel. Rotation symbol indicates alternative view used for secondary binding mode observed in a subset of models where c-Myc interacts with the backside of the kinase domain distal from the active site. **d)** Predicted alignment error (PAE) plots from three representative runs.


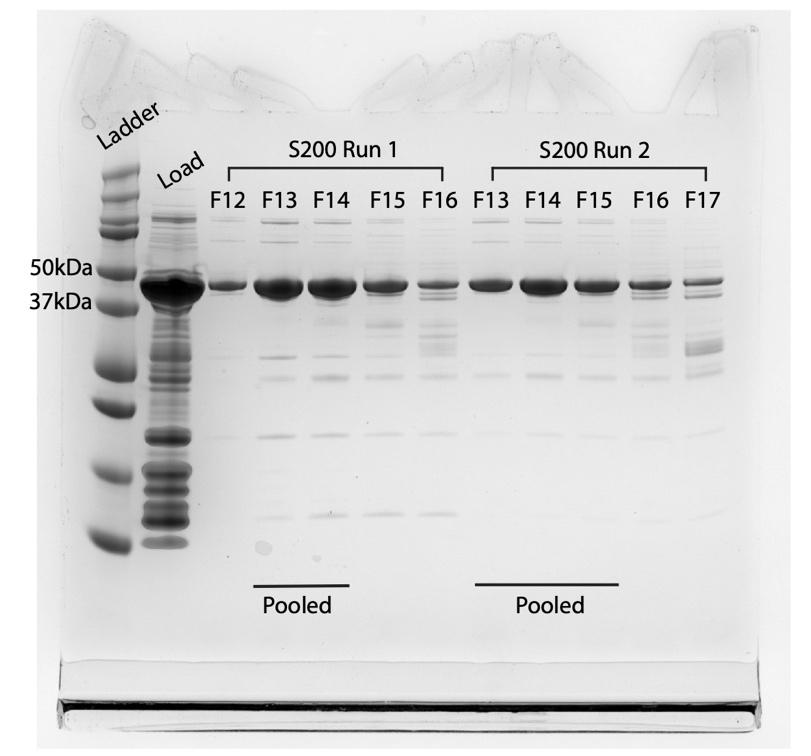


**Figure S14.** **SDS-PAGE of final purification step for c-Myc^1-331^ C117.**

A representative SDS-PAGE is shown for the final gel filtration step from the purification of c-Myc^1-331^ C117. Two separate runs were performed and the indicated fractions from both were pooled.
